# Sequence-dependent attack on peptides by photoactivated platinum anticancer complexes†
†Electronic supplementary information (ESI) available: Detailed MS data and discussion of effects of Pt on MS/MS fragmentation, as Scheme S1, Tables S1–S14 and Fig. S1–S10. See DOI: 10.1039/c7sc05135b


**DOI:** 10.1039/c7sc05135b

**Published:** 2018-02-12

**Authors:** Christopher A. Wootton, Carlos Sanchez-Cano, Andrea F. Lopez-Clavijo, Evyenia Shaili, Mark P. Barrow, Peter J. Sadler, Peter B. O'Connor

**Affiliations:** a Department of Chemistry , University of Warwick , Gibbet Hill Road , Coventry CV4 7AL , UK . Email: p.oconnor@warwick.ac.uk ; Email: p.j.sadler@warwick.ac.uk ; Fax: +44 (0)24 76151009 ; Fax: +44 (0)24 765 23819 ; Tel: +44 (0)24 76151008 ; Tel: +44 (0)24 765 23818

## Abstract

Octahedral platinum(iv) complexes such as *trans*,*trans*,*trans*-[Pt(N_3_)_2_(OH)_2_(pyridine)_2_] (**1**) are stable in the dark, but potently cytotoxic to a range of cancer cells when activated by UVA or visible light, and active *in vivo*. Photoactivation causes the reduction of the complex and leads to the formation of unusual Pt(ii) lesions on DNA. However, radicals are also generated in the excited state resulting from photoactivation (J. S. Butler, J. A. Woods, N. J. Farrer, M. E. Newton and P. J. Sadler, *J. Am. Chem. Soc.*, 2012, **134**, 16508–16511). Here we show that once photoactivated, **1** also can interact with peptides, and therefore proteins are potential targets of this candidate drug. High resolution FT-ICR MS studies show that reactions of **1** activated by visible light with two neuropeptides Substance P, RPKPQQFFGLM-NH_2_ (**SubP**) and [Lys]^3^-Bombesin, pEQKLGNQWAVGHLM-NH_2_ (**K^3^-Bom**) give rise to unexpected products, in the form of both oxidised and platinated peptides. Further MS/MS analysis using electron-capture dissociation (ECD) dissociation pathways (enabling retention of the Pt complex during fragmentation), and EPR experiments using the spin-trap DEPMPO, show that the products generated during the photoactivation of **1** depend on the amino acid composition of the peptide. This work reveals the multi-targeting nature of excited state platinum anticancer complexes. Not only can they target DNA, but also peptides (and proteins) by sequence dependent platination and radical mechanisms.

## Abstract 2

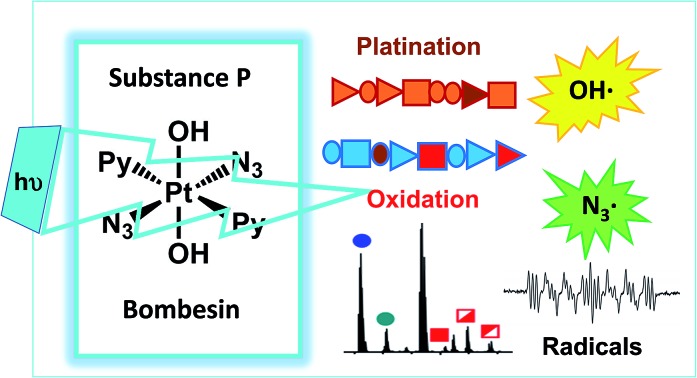
Octahedral anticancer platinum(iv) complexes such as *trans*,*trans*,*trans*-[Pt(N_3_)_2_(OH)_2_(pyridine)_2_] (**1**) can target peptides (and proteins) by sequence-dependent platination and radical mechanisms when activated by UVA or visible light; the specific products are highly dependent on their amino acid composition of the peptide.

## Introduction

Diazido platinum(iv) complexes such as *trans*,*trans*,*trans*-[Pt(N_3_)_2_(OH)_2_(pyridine)_2_] (**1**) are promising agents for use in Photo-Activated Chemotherapy (PACT),^1^,^2^ which offers potential temporal and spatial control over their activity,^3^–^5^ reducing side effects through unwanted attack on normal tissues.^6^,^7^

**Scheme 1:**
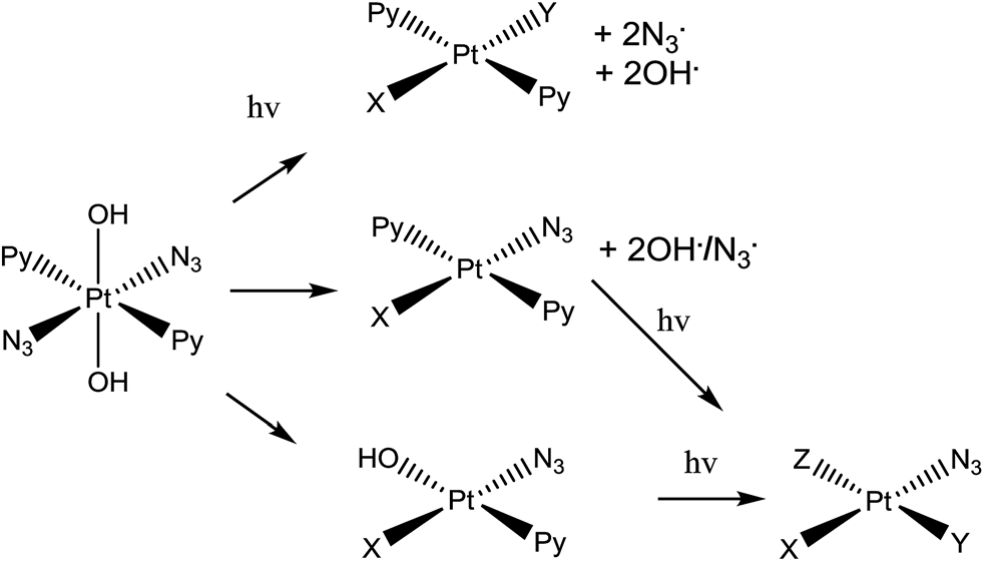
*Trans*,*trans*,*trans*-[Pt(N_3_)_2_(OH)_2_(py)_2_] (complex **1**), the photo-activatable prodrug used in this study, along with illustrative photo-decomposition pathways leading to Pt^II^ products which can form bonds/inter-strand cross-links on DNA.^1^

Complex **1** (scheme 1) and its analogues exhibit little activity in the dark, yet possess potent antiproliferative activity *in vitro* against a wide range of cancer cells upon irradiation with visible light,^8^ and are also active *in vivo* towards oesophaegeal cancer after short time exposures to blue light.^9^ Furthermore, they do not rely on the conversion of ^3^O_2_ to ^1^O_2_ to kill cancer cells, as sensitisers used in photodynamic therapy (*e.g.* based on tetrapyrroles),^10^ which potentially can allow them to function effectively under the hypoxic conditions found in tumors.^7^,^11^,^12^

Complex **1** is more active than its *cis* isomer, and not cross resistant with cisplatin.^8^ Its mechanism of action seems to involve the platination of DNA by Pt(ii) photoproducts, forming inter-strand DNA cross-links, which are more difficult to repair than DNA-adducts formed by *cis*-analogues.^11^ However, radicals are also generated during irradiation of the complex with visible light.^1^,^6^,^13^,^14^ Initially it was believed that the mechanism of photodecomposition of **1** simply involved excitation of the ligand(azide)-to-metal (Pt^IV^) charge-transfer band, with one-electron transfer from each azide ligand giving rise to Pt^II^ and two azidyl radicals which can combine to form three molecules of N_2_.^1^ However, the detailed photodecomposition pathways are complex, can lead to the production of azidyl radicals, singlet oxygen (even in absence of gaseous O_2_), nitrenes, and hydroxyl radicals,^1^,^6^,^13^,^14^ and depend strongly on the biomolecules present. For example, loss of N_2_ from a bound azide can lead to nitrene intermediates which might attack thioethers such as the side-chain of methionine.^15^ Additionally, the production of azidyl radicals is quenched in the presence of tryptophan (but not other amino acids such as glycine or tyrosine).^14^ This suggests that **1** might interact with peptides and proteins inside cells, following different photodecomposition pathways depending on their sequences. However, the interaction of photoactivated **1** with peptides has yet to be studied.

Tandem mass spectrometry (MS/MS) analysis has been successful in studying an array of peptides and proteins while retaining vital post-translational modifications (PTM's).^16^–^18^ This technique has been used to investigate the interactions of biomolecules with platinum drugs such as cisplatin,^19^–^21^ transplatin,^22^ oxaliplatin,^23^ or diiodido–platinum complexes,^24^ but also ruthenium,^25^–^28^ and organometallic iridium and osmium complexes.^29^,^30^ Electron-based dissociations are particularly useful when studying metallodrug interactions where the top-down approach has allowed the characterisation of entire protein sequences and the unambiguous determination of multiple binding sites for metallodrugs on various proteins,^21^,^31^–^33^ without further chemical modifications such as digestion which can disrupt/dissociate modifications. Electron capture dissociation (ECD) MS/MS is particularly effective for PTM analysis due to its non-ergodic character^34^ and its ability to fragment species without loss of fragile PTM's.^35^

Herein, we use ultra-high resolution Fourier Transform Ion Cyclotron Resonance Mass Spectrometry (UHR-FT-ICR MS) to study the interaction of *trans*,*trans*,*trans*-[Pt(N_3_)_2_(OH)_2_(pyridine)_2_] (**1**) with two model peptides (neuropeptides); Substance P, RPKPQQFFGLM-NH_2_ (**SubP**), and [Lys]^3^-Bombesin, pyrQKLGNQWAVGHLM-NH_2_ (**K^3^-Bom**). Both of which have amidated C-termini. Our experiments show for the first time that photoactivation of **1** leads to both amino-acid-specific platination and oxygenation of peptides.

## Results and discussion

**K^3^-Bom** and **SubP** are naturally occurring peptides with a role in some types of cancer; **K^3^-Bom** is a known tumour marker,^36^ and **SubP** has elevated levels in several types of cancer cells.^37^ Initial experiments showed that the use of bright red light during sample preparation and analysis had no effect on **1**, while low levels of white light induced the photoactivation of the complex. Therefore, all samples were prepared and analysed in darkness or with very low levels of red light, to avoid activation of **1** prior to irradiation with blue light.

Photoactivation of **1** in presence of **SubP** or **K^3^-Bom** was first followed using UV-vis spectroscopy (Fig. S1†). Complex **1** exhibits an intense azide-to-Pt(iv) charge-transfer band (*λ*_max_ = 295 nm) which can be used to monitor the photodecomposition of the complex.^1^**SubP** had little apparent effect on the rate of decomposition of **1**. However, **K^3^-Bom** slowed this process, and induced the formation of a new peak at *ca.* 250 nm.

**Fig. 1:**
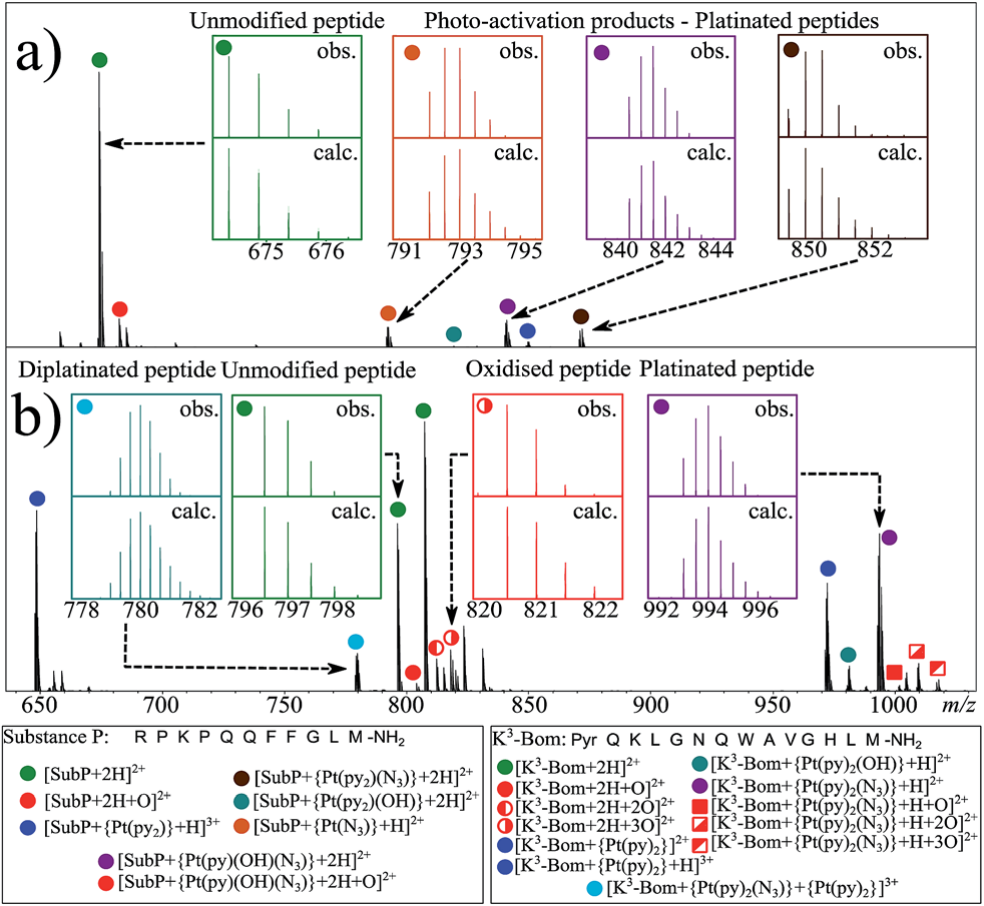
nESI FT-ICR mass spectra of (a) *ca.* 1 μM aqueous complex **1+SubP**, and (b) complex **1+K^3^-Bom** reaction mixtures (0.5 : 1 drug : peptide ratio) after 1 and 2 h of irradiation (respectively) with blue visible light (463 nm). Inset; various isotopic patterns for the observed (obs.) and calculated (calc.) species assigned from the mass spectra – showing the influence of platinum on observed isotopic distributions. Green filled circles indicate unmodified peptide species, red indicates oxidised species (both peptide and platinated peptides), other colours indicate platinated peptide species with different Pt(ii)-based modifications.

Using nESI-FT-ICR-MS, we analysed reaction mixtures of **1+SubP** and **1+K^3^-Bom** (0.5–1 drug : peptide) irradiated with blue light (463 nm; 30–120 min) to activate **1**. Peaks for a total of 8 and 12 products were assigned for the reactions of **1+SubP** and **1+K^3^-Bom**, respectively (Fig. 1, Table S2†). Most can be assigned to different platinated adducts of the peptides. For the reaction between **1+SubP**, only mono-platinated adducts were observed, containing the modifications {Pt(py)(OH)(N_3_)}, {Pt(py)_2_(N_3_)}, {Pt(N_3_),}, and {Pt(py)_2_} with reasonable intensity, and {Pt(py)_2_(OH)} at low intensity. However, analysis of the reaction **1+K^3^-Bom** showed both mono- and di-platinated adducts, containing {Pt(py)_2_}, {Pt(py)_2_(N_3_)}, {Pt(py)_2_(OH)}, and both {Pt(py)_2_} and {Pt(py)_2_(N_3_)} modifications simultaneously.

Interestingly, both unmodified and platinated adducts of the peptides were found to be oxidised when irradiated in presence of **1**. **SubP** species had the addition of one oxygen (by exact mass), while up to three oxygen atoms were found on **K^3^-Bom** species. Similar species were not observed upon irradiation of the peptides in absence of **1**, or when **1** + peptide mixtures where not irradiated with blue light.

### Photoactivated platination of [Lys]^3^-Bombesin

Platinum-containing **K^3^-Bom** adducts were readily identified in MS and MS/MS spectra by platinum's characteristic isotopic pattern (Fig. 1, inset). Clear peaks for various platinated species can be observed simultaneously, showing release of 1-2 hydroxido ligands and 1-2 azido ligands. Retention of the pyridine ligands during the photoactivation of **1** correlates well with previous ^1^H-NMR experiments on model reactions with guanine derivatives,^6^,^11^ as do products in which one azido ligand remains bound in the case of the {Pt(py)_2_(N_3_)}^+^ modification.^6^

**Fig. 2:**
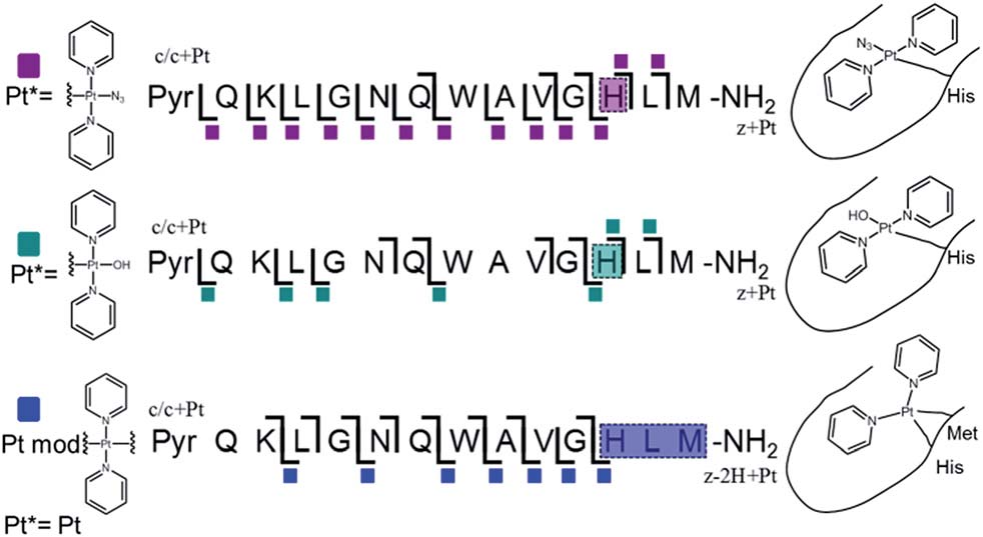
Platinated **K^3^-Bom** peptide species observed and fragmented by ECD MS/MS; coloured squares indicate a modification observed on the particular fragment. These modifications can be readily assigned to single amino acid residues. Fully annotated spectra are shown in Fig. S2.†

ECD MS/MS analysis of the platinated **K^3^-Bom** species is summarised in Fig. 2. Full assignment lists and ECD MS/MS spectra are in Tables S2–S4 and Fig. S2.† **K^3^-Bom** contains methionine, histidine and lysine residues, all potential S or N donor ligands. Pt(ii) complexes are known to bind preferentially at the S of Met, followed by His N.^20^,^32^,^38^–^40^ Pt–S bonds are usually strong, and survive harsh pH digestions and high energy gas phase dissociations in tandem MS experiments.^20^ However, {Pt(py)_2_(N_3_)}^+^ and {Pt(py)_2_(OH)}^**+**^ modifications from mono-platinated **K^3^-Bom** adducts were unambiguously bound at the His^12^ residue, despite the C-terminal Met.^14^ MS/MS analysis of [**K^3^-Bom**+Pt(py)_2_]^2+^ species yielded no sequence-informative fragments, only ligand and/or amino acid side chain loss peaks. Such behaviour has been attributed to extended cyclic structures which can disrupt usual MS/MS fragmentation.^41^ However, the presence of metal centres presents unique challenges to MS and MS/MS peptide/protein analysis.^41^–^44^

The charged Pt centre altered the ECD MS/MS fragmentation pattern by providing a fixed charge at the point of interaction (*e.g.* His^11^ of **K^3^-Bom**), but also enhanced side chain losses of amino acid groups present. Electron capture at the Pt^II^ centre/electron transfer of previously captured electrons on the peptide also caused additional ligand loss from the bound complex and additional fragmentations from amino acid residues, including unique side chain losses from methionine residues (Scheme S1†). CAD MS/MS of platinum complex-containing species caused gas-phase dissociation of the platinum-bound ligands, even at low energies (<5–7 V), creating a reactive Pt^II^ centre, which quickly cyclised with available peptide groups and produced uninformative fragmentation spectra.

Still, ECD MS/MS of [**K^3^-Bom**+Pt(py)_2_+H]^3+^ species produced abundant backbone fragmentation (Fig. S2c†). This showed simultaneous binding at His^12^ and Met^14^ residues of {Pt(py)_2_} photoproducts, producing a small cyclic region in the modified peptide species.

### Photoactivated platination of substance P

**Fig. 3:**
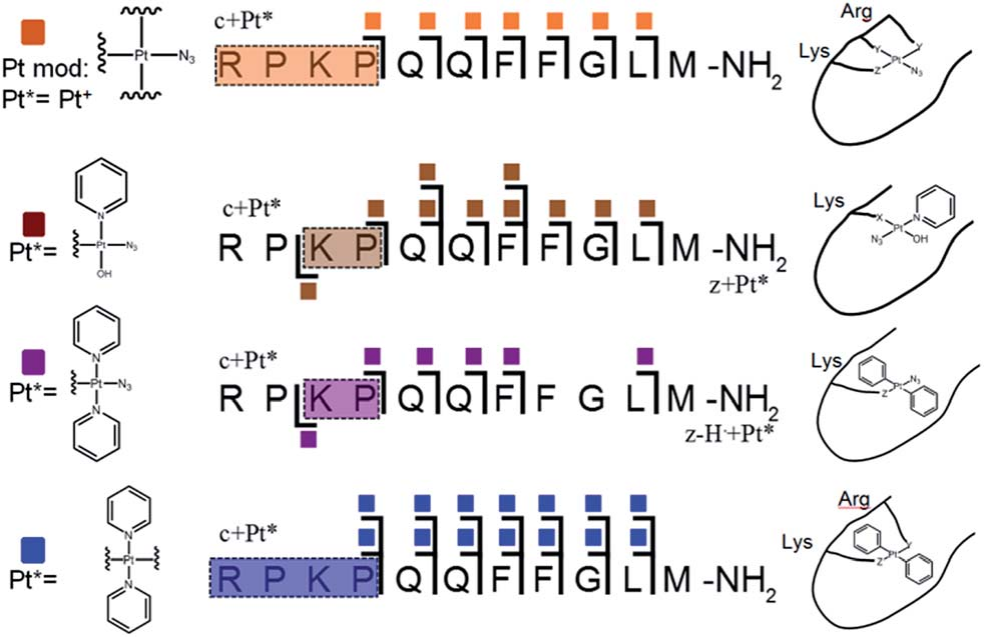
Platinated **SubP** peptide species observed and fragmented by ECD MS/MS; coloured squares indicate a modification on the particular fragment. Fully annotated spectra are shown in Fig. S3.†

Unlike **K^3^-Bom**, **SubP** contains no histidine residues, and photoactivation of **1+SubP** mixture led to loss of a pyridine ligand, producing ({Pt(py)(OH)(N_3_)} as a major peptide modification; Fig. 1a and 3). This suggests that all the types of ligands found in **1** can be released during photoirradiation, and highlights the importance of peptide sequence in determining the pathways followed during photodecomposition.

ECD MS/MS shows that [**SubP**+{Pt(py)(OH)(N_3_)}+2H]^2+^, [**SubP**+{Pt(py)_2_(N_3_)}+H]^2+^, [**SubP**+{Pt(N_3_)+H]^2+^, and [**SubP**+{Pt(py)_2_+H}]^3+^ species are platinated within the four N-terminal amino acids of the peptide (Fig. 2, Tables S5–7, Fig. S3†). [**SubP**+{Pt(py)_2_(OH)}+H]^2+^ species were observed only at very low intensity at all irradiation times, and reliable MS/MS fragmentation could not be obtained. ECD induced fragmentation of the N-terminal region of **SubP** (RPKP) is restricted by proline residues,^45^ and the overall structure is held together *via* the “proline effect”.^46^ This hampered determination of the platination sites of **SubP**. However, the fragmentation allowed the binding site for **1** to be located between Lys^3^ and Pro.^4^

Interestingly, spectra from **1+K^3^-Bom** mixtures after different irradiation times (30, 60, 90 or 120 min) always contained the same photoproducts, increasing their overall, but not their relative intensity at longer irradiation times. However, irradiation of **1+SubP** mixtures for different times showed preferential formation of different products at different times. [**SubP**+Pt(py)(OH)(N_3_)+2H]^2+^ was the major product at short irradiation times, while longer irradiations of **1+SubP** mixtures led to further ligand release, with [**SubP**+Pt(N_3_)+H]^2+^ species increasing over time. This suggests that photoreactions of **1** can continue after the formation of Pt(ii) species.

### Methionine and tryptophan oxidation

**Fig. 4:**
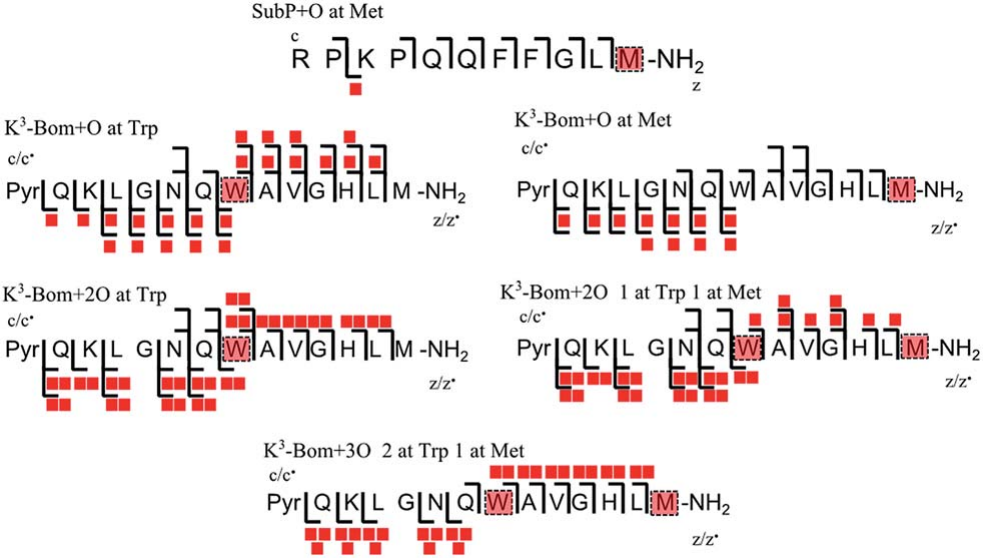
Oxidised peptide species observed and fragmented by ECD MS/MS. Oxidative modifications (indicated by red squares) can be readily located on single amino acid residues (highlighted). Fully annotated tandem mass spectra can be found in the ESI (Fig. S5†).

In addition to peptide platination; an array of oxidised products was also observed following photoactivation of **1**. Oxidised peptide species (*i.e.* [**SubP**+O+2H]^2+^, [**K^3^-Bom**+O+2H]^2+^, [**K^3^-Bom**+2O+2H]^2+^, and [**K^3^-Bom**+3O+2H]^2+^) were further studied using FT-ICR-MS and ECD MS/MS (Fig. 4 and S5, Tables S8–S11†). Fragmentation maps revealed that oxidation of the peptides occurs at Met^11^ for **SubP** and Met^14^ and Trp^8^ for **K^3^-Bom**. Methionine residues in both peptides can be oxidised to the sulfoxide,^47^ and further to sulfone species.^48^ However, MS/MS shows that photoactivation of **1** in presence of the peptides **K^3^-Bom** and **SubP** led only to the formation of sulfoxide species.

**Scheme 2:**
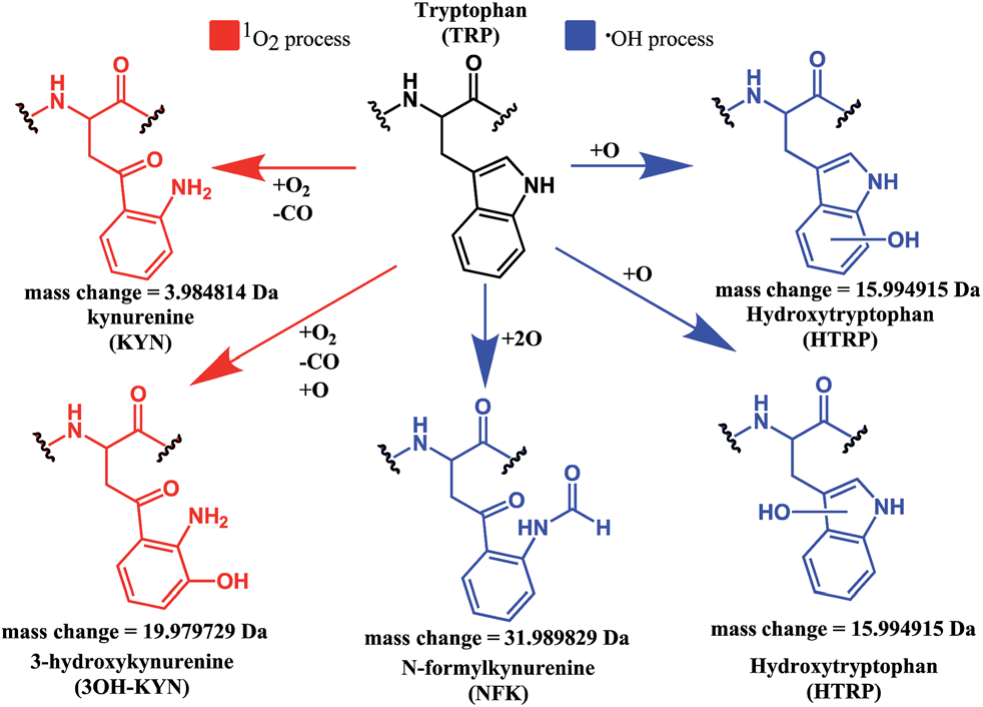
Possible oxidation products arising from ^1^O_2_ (KYN and 3OH-KYN) and radical (HTRP and NFK) oxidation of a tryptophan side-chain^50^,^51^ along with corresponding calculated mass changes for the modifications. Singlet oxygen induced processes are shown in red, while those induced by hydroxyl radical processes are shown in blue.

Both mono- and di-oxidised **K^3^-Bom** species showed a two-product fragment distribution, relating to oxidation at Met^14^ or Trp^8^ for mono-oxidised species, and to single oxidation of both Met^14^ and Trp^8^ residues, or double oxidation of Trp^8^ for di-oxidised species (Fig. 4, Tables S9–11, Fig. S5b–c†). [**K^3^-Bom**+3O+2H]^2+^ species showed both oxidation at Met^14^ and double oxidation of Trp^8^ residues. Tryptophan oxidation is known to give rise to a variety of side-chain modifications, which are dependent of the nature of the oxidising agent.^49^,^50^ Hydroxy-tryptophan (HTRP) and *N*-formyl kynurenine(NFK) are formed when the 5-membered indole ring of tryptophan is attacked by hydroxyl radicals, while kynurenine (KYN) and 3-hydroxy-kynurenine (3OH-KYN) arise from singlet oxygen-induced oxidation (Scheme 2). These reaction products have different mass changes compared to the original amino acid, and can be used to provide insights into the mechanism of the oxidation itself.^50^

UHR-FT-ICR MS spectra of **1+K^3^-Bom** mixtures (Fig. 1b) and the corresponding MS/MS spectra (Fig. S2a–d†) show clearly that tryptophan oxidation leads to the formation of HTRP and NFK for the mono and di-oxidised species, respectively (+15.994915 Da and +31.989829 Da mass shifts). This suggests that hydroxyl radicals are generated when **1** is photoactivated in the presence of **K^3^-Bom**, and are responsible for the oxidation of methionine and tryptophan residues. Electron transfer from an axial hydroxido ligand and an azido ligand to Pt^IV^ can result in retention of one N_3_ ligand in the Pt^II^ photo-product (Fig. 1).

### Detection of radicals

**Fig. 5:**
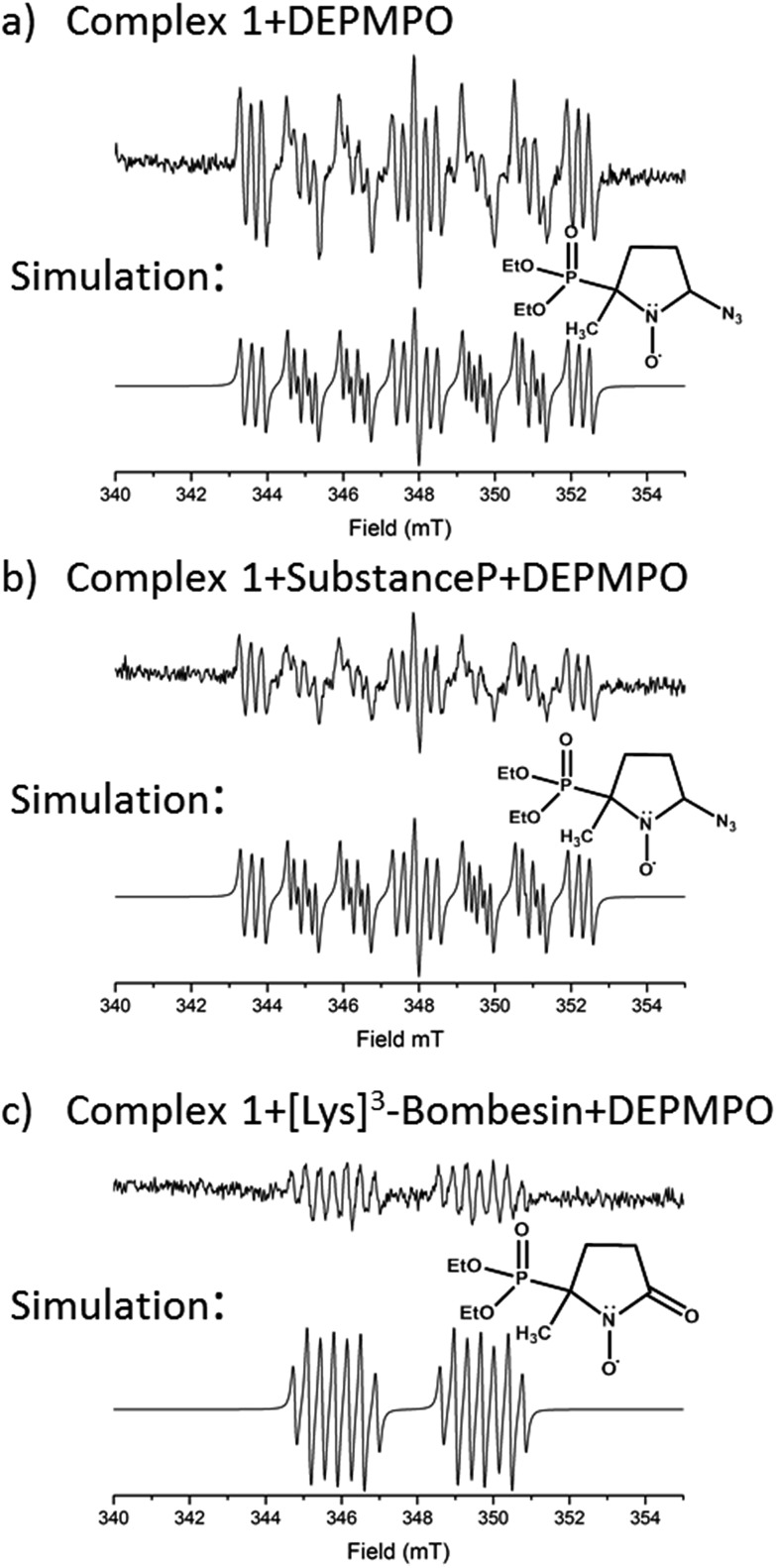
EPR spectra showing the radicals trapped by DEPMPO during the photoactivation (with blue visible light) of (a) **1** (b) **1+SubP** and (c) **1+K^3^-Bom**. Inset shows simulations of corresponding spectra and the structures of the spin-trapped azidyl radicals (a + b) and oxidised DEPMPO arising from attack by the high concentration of OH radicals produced *via* photoactivation of **1** with **K^3^-Bom** (c).^52^

EPR was used to trap radicals generated during the photoactivation of **1**, **1+SubP**, and **1+K^3^-Bom** with DEPMPO as the spin trap (Fig. 5, Table S12, Fig. S6†). Azidyl radicals were trapped during the photoactivation of complex **1** alone, and also from **1+SubP** mixtures. However, the EPR spectra obtained from irradiation of **1+K^3^-Bom** mixtures were dramatically different.

Oxidised adducts of the DEPMPO trap were observed (DEPMPOX, Fig 5c). Such species can be formed by oxidation of DEPMPO-OH adducts,^52^ and arising from the attack by hydroxyl radicals. These studies therefore confirm that the photoreaction pathways for complex **1** can therefore not only involve electron transfer from azide ligand to Pt^IV^ but also from an hydroxido ligand to form N_3_˙ and HO˙ radicals.

### Effect of free tryptophan on peptide oxidation

Reaction mixtures containing **SubP+1+L-Trp** and **K^3^-Bom+1+L-Trp** (mol ratio 2 : 1 : 0.13 (peptide : drug : Trp); 31 μM Trp) were irradiated with blue light (463 nm) for 60 and 120 min, respectively, min and monitored by nESI-FT-ICR-MS (Table S13, Fig. S7†). Addition of Trp had little effect on the type of species produced and peptide platination observed in the photoreaction of **SubP+1**. However, Trp reduced the level of peptide oxidation by 29% and 95% for non-platinated and platinated-oxidised species, respectively. Trp affected more dramatically the photoreaction of **K^3^-Bom+1** mixtures. Every oxidised and platinated species was reduced in intensity (except [**K^3^-Bom**+O]^2+^ and [**K^3^-Bom**+3O]^2+^) by 32–98%. This suggests that the Trp residue in **K^3^-Bom** itself could play a role in determining the extent of platination of the peptide.

## Conclusions

These studies show for the first time that photoactivation of the diazido Pt^IV^ anticancer prodrug **1** can readily lead to a dual attack on peptide amino acids, either oxidation, or platination, or both. Additionally, the amino acid composition can have a dramatic effect on determining the course of the reactions. Both **SubP** and **K^3^-Bom** were oxidised, platinated, and oxidised and platinated on photoactivation of complex **1**, but the nature of the bound photoproducts of **1** and the amino acid residues modified depend on the amino acid composition of the peptide. His^12^ of **K^3^-Bom** was preferentially platinated, and both pyridine ligands were retained in all the photoproducts observed, whereas for **SubP**, a different range of products was observed, and complex **1** released one or two pyridine ligands with apparent binding to Lys,^3^ and Arg^1^ residues. Photoactivation led to the oxidation of specific sites in both peptides. For **SubP** oxidation occurred only at Met,^11^ whereas for **K^3^-Bom**, up to three stages of oxidation were observed: oxidation at Met^14^ and a single or double oxidation of the Trp^8^ residue. This is the first observation of Met and Trp oxidation by this class of photoactivatable platinum anticancer complexes. Trp modification yielded hydroxytryptophan (HTRP) and N-formylkynurenine (NFK) species (Scheme 2), indicating that hydroxyl radicals are responsible for the oxidative attack. Interestingly, oxidation of Trp to NFK is also a critical step in the biological production of NAD^+^. The dependence of radical generation on the nature of the peptide was further confirmed by EPR spin-trapping.

The role of tryptophan as a redox-active amino acid,^53^ and the formation of HTRP and NFK species suggest that radical-based processes might play an important role in the mechanism of action of this class of Pt^IV^ photoactivated complexes. Such production of oxidative stress may provide some selectivity for attack on cancer cells *versus* normal cells, since cancer cells have malfunctioning mitochondria and are particularly susceptible to redox stress.^54^–^56^

## Experimental

Substance P, [Lys]^3^-bombesin, and formic acid were purchased from Sigma Aldrich Company Ltd., Dorset, UK. Low concentration Agilent tuning mix was purchased from Agilent Technologies (Santa Clara, CA). Complex **1** was synthesised and characterised as described elsewhere.^6^ EPR tubes were purchased from Wilmad Labglass. The spin trap 5-(diethoxyphosphoryl)-5-methyl-1-pyrroline-*N*-oxide (DEPMPO) was obtained from Enzo Life Sciences in high purity. Ultra-pure water was obtained from a Milli-Q UV III system (Milli-Q, Hertfordshire, UK).

### Reactions of peptides with **1**

Aliquots of aqueous solutions of Substance P (1 mM) and [Lys]^3^-Bombesin (1 mM) were mixed with an aqueous solution of **1** (250 μM) to give solutions of 0.5 : 1 drug : peptide mol ratio. The samples were then irradiated under 463 nm (blue visible) light at 298 K for various times, before being diluted to MS concentrations (*ca.* 1 μM) and either analysed immediately or frozen at –80 °C prior to MS analysis. Freshly prepared samples were compared to those frozen for one to several weeks and showed no observable variation in the mass spectra obtained. Reactions involving tryptophan were carried out using similar procedures as above together with the addition of aqueous tryptophan solution (1 mM) to peptide + **1** solutions to achieve a mol ratio of 0.5 : 1 : 0.125 (drug : peptide : tryptophan), *i.e.* concentration of tryptophan was 1/8^th^ of drug concentration, as used previously by Butler *et al.*^14^

### FT-ICR mass spectrometry

Nano-electrospray (nESI) mass spectrometry was performed on a Bruker SolariX Fourier Transform Ion Cyclotron Resonance Mass spectrometer (FT-ICR MS) fitted with a 12 tesla actively shielded magnet (Bruker Daltonics, Bremen, Germany). Aqueous peptide samples (1 μM) were spiked with 0.3% formic acid (v/v) to aid ionisation during nESI. Solutions containing **1** (including reaction mixtures) were analysed *via* nESI in Milli-Q water with no added acid.

For ECD MS/MS analysis; the species of interest were isolated in the first quadrupole, externally accumulated in the collision cell for 0.1–7 s and then transferred to the infinity cell for Electron Capture Dissociation (ECD) fragmentation and detection. Ions in the infinity cell were irradiated with 1.3–1.6 eV electrons from a 1.5 A hollow cathode dispenser for 50–600 ms prior to detection.

MS/MS spectra were internally calibrated using the minimal number of unmodified (peptide spectra) or modified (Pt adduct spectra) c/z ions and the charge reduced species [M + *n*H]^*n*–1+^˙ where possible (species used for calibration are marked). A dual-spray nESI experiment was also conducted using the [**K^3^-Bom**+2H]^2+^ ion and ions from Agilent Tune mix, utilising an in-cell isolation (Multi-CHEF)^57^ and identical ECD parameters to validate the internal calibration of the MS/MS spectra. Similar standard deviations were found for the ECD fragmentation spectra when calibrated with either fragment ions or with tune-mix peaks (see ESI Fig. S8 and Table S14†). This dual-spray Multi-CHEF ECD approach was also used for some **1** + peptide reaction product ECD spectra to improve the internal calibration (spectra and calibration peaks marked accordingly).

### Electron paramagnetic resonance (EPR)

EPR spectra were recorded at ambient temperature on a Bruker EMX (X-band) spectrometer fitted with a cylindrical TM110 mode cavity (Bruker 4103TM). Samples were contained in a quartz capillary tube (I.D. 1.0 mm; O.D. 1.2 mm; Wilmad Labglass) sealed with T-Blu Tac®, placed inside larger quartz tubes (O.D. 2.0 mm) to achieve easy and accurate positioning of the sample inside the resonator. Typical key EPR spectrometer settings were modulation amplitude 2.0 G, microwave power 0.63 mW, 1.0 × 10^5^ receiver gain, conversion time 81.92 ms, time constant 81.92 ms, sweep width 200 G, and a repeated number of 10 X-scans with a resolution in *Y* of 5 or 9. Spin-trapping experiments were performed on aqueous solutions of the complex with excess of spin trap (1 mM **1**, 6 mM DEPMPO) in the presence or absence of peptides (1 mM). A visible blue light emitting diode (LED, *λ* = 463 nm, 64 mW cm^–2^) was used as the source of irradiation and placed at a distance of 8.5 cm from the tube in the EPR cavity. Irradiations lasted up to 2 h, each slice corresponding to 14 min of irradiation (10 scans). The TM110 EPR cavity used is equipped with a grid on one side allowing optical access (*ca.*, 50%) transmission. The refractive index of quartz is approximately 1.55; hence at normal incidence approximately 5% of the incident light is reflected at an air quartz interface. EPR spectra were analysed and simulated using the EASYSPIN software.^58^

### UV-vis spectroscopy

Aqueous solutions of **1** (60 μM), **SubP** (120 μM), **K^3^-Bom** (120 μM), **1** (60 μM) + **SubP** (120 μM), and **1** (60 μM) + **K^3^-Bom** (120 μM) were prepared and irradiated as above, then studied using a Cary 300 scan UV-visible spectrophotometer (Agilent, California, US). Solutions were analysed in quartz cuvettes (0.5 mL, 1 cm path length), with scanning in the region of 200–800 nm at a rate of 10 nm s^–1^ (600 nm min^–1^), average time = 0.1 s, data interval 1 nm.

## Conflicts of interest

There are no conflicts to declare.

## Supplementary Material

Supplementary information
