# Supplementary material for: Sequence-dependent attack on peptides by photoactivated platinum anticancer complexes
Source: Chem Sci. 2018 Feb 12;9(10):2733–9. doi: 10.1039/c7sc05135b (PMC5911824; doi:10.1039/c7sc05135b)
Supplement: Supplementary file 1 [file SC-009-C7SC05135B-s001.pdf]

Electronic Supplementary Material (ESI) for Chemical Science.
This journal is © The Royal Society of Chemistry 2018

Sequence-Dependent Attack on Peptides by Photoactivated Platinum Anticancer Complexes

Christopher A. Wootton, Carlos Sanchez-Cano, Andrea F. Lopez-Clavijo, Evyenia Shaili, Mark P. Barrow, Peter J. Sadler, * and Peter B. O'Connor *

Electronic Supplementary Information

Figure S1. UV-vis spectra of (a) complex **1**, (b) + Substance P, (c) + $[Lys]^3$-Bombesin, on irradiation with blue light for the times shown (min, decreasing absorbance at 300 nm).

Table S1. Species observed in the nESI-FT-ICR mass spectra of solutions of complex **1** + (A) SubP and (B) K^3-Bom, post-photoactivation.

Figure S2. ECD MS/MS spectra of platinated K^3-Bom species individually isolated and dissociated using ECD within the FT-ICR MS a) $[K^3\text{-Bom}+Pt(py)_2(N_3)+H]^{2+}$ b) $[K^3\text{-Bom}+Pt(py)_2(OH)+H]^{2+}$ c) $[K^3\text{-Bom}+Pt(py)_2]^{2+}$ d) $[K^3\text{-Bom}+Pt(py)_2+H]^{3+}$.

Figure S3. ECD MS/MS spectra of platinated Substance P species individually isolated and dissociated using ECD within the FT-ICR MS a) $[SubP+Pt(N_3)+H]^{2+}$ b) $[SubP+Pt(py)(OH)(N_3)+H]^{2+}$ c) $[SubP+Pt(py)_2(N_3)+H]^{2+}$ d) $[SubP+Pt(py)_2+H]^{3+}$.

Tables S2-S7. Assignments for individual ECD MS/MS spectra of the isolated platinated peptide species.

Figure S4. (a) ECD MS/MS spectra of di-platinated K^3-bom species, and (b) expansion of side chain loss (SCL) region resulting from electron capture at a platinum centre, causing ligand loss.

Figure S5. ECD MS/MS spectra of oxidised peptides individually isolated and dissociated using ECD within the FT-ICR MS. a) Substance P+O. b) K^3-Bom+O. C) K^3-Bom+2O. d) K^3-Bom+3O.

Tables S8-S11. Aassignments for individual ECD MS/MS spectra of the isolated oxidised peptide species. **S8**: Substance P+O; **S9**: K^3-Bom+O; **S10**: K^3-Bom+2O; **S11**: K^3-Bom+3O.

Table 12. EPR data for irradiation with blue visible light of (A) complex **1** in the presence of the spin-trap DEPMPO, (B) +SubstanceP, (C) +$[Lys]^3$-Bombesin.

Figure S6. X-band EPR spectra at various times after irradiation with blue light of complex 1 + DEMPO + (a) no peptide, (b) SubP (c) K^3-Bom. Simulations show spectra for adducts (a) DEMPO-N_3, (b) DEMPO-N_3, (c) DEMPOX.

Table 13. Relative intensities of peaks for assigned species in the FTMS of irradiated complex **1**+ (A) Substance P, and (B) K^3-Bom reaction mixtures, with and without added tryptophan.

Figure S7. Bar charts showing the relative intensity changes for MS peaks for the products of reactions between **1**+peptide and **1**+peptide+Trp for both **SubP** (top) and **K^3-Bom** (bottom).

Figure S8. (a) nESI-FT-ICR MS spectrum of (a) Substance P and (b) K^3-Bom; (c) ECD MS/MS spectrum of K^3-Bom

Table S14. Peptide fragment assignments and associated mass errors for the ECD MS/MS spectra of the $[K^3\text{-Bom}+2H]^{2+}$ species with and without the use of co-isolated calibrant ions.

Figure S9. ECD MS/MS of unmodified Substance P (top), a list of assignments (middle), and corresponding fragmentation map (bottom)

Effects of platinum on MS/MS fragmentation

Scheme S1: Platinum-centred side chain losses from methionine commonly observed during ECD MS/MS studies of platinated peptides.

Scheme S2. Proposed mechanism for the loss of the Pt(II) modification from the peptide during ECD; ligands shown vary with the nature of the Pt adduct.

References

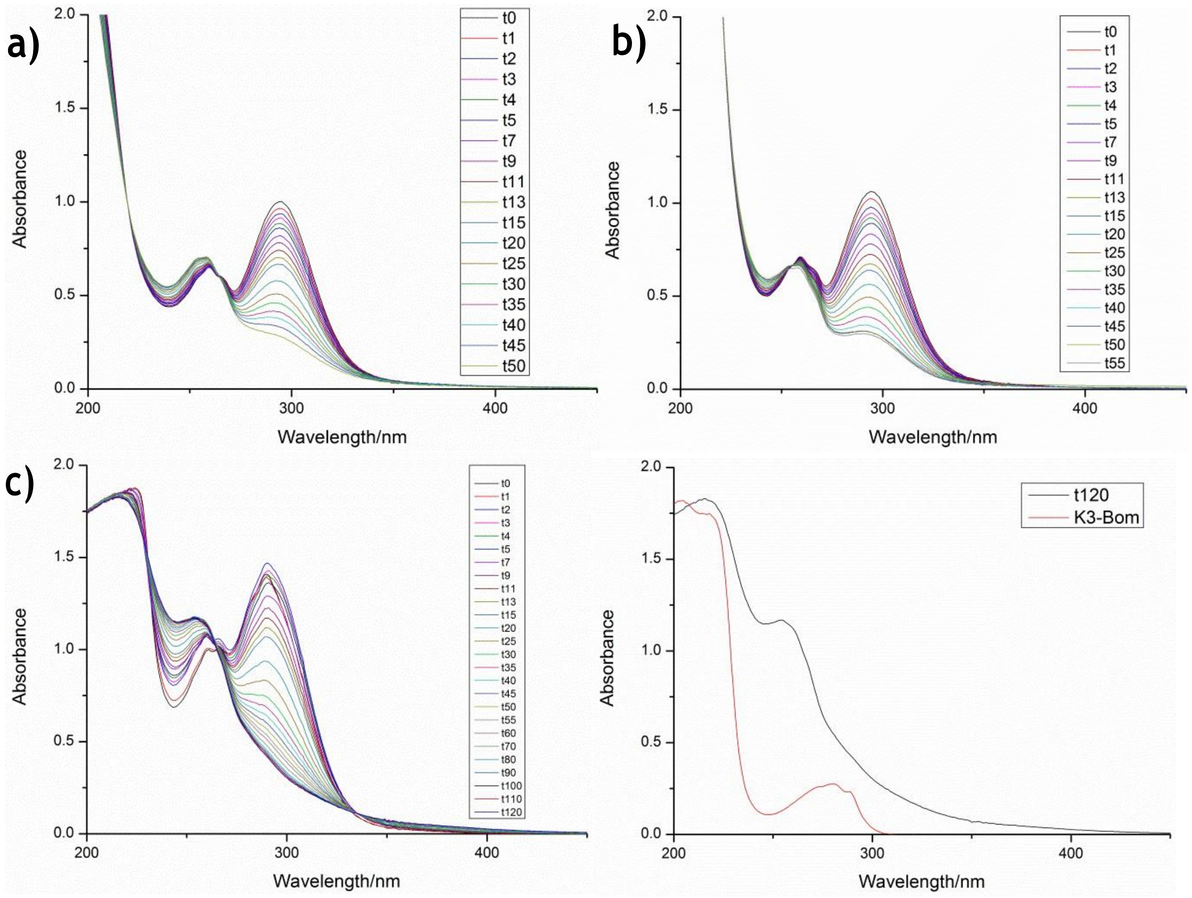

Figure S1. UV-vis spectra of (a) complex **1**, (b) + Substance P, (c) + $[Lys]^3$-Bombesin, on irradiation with blue light for the times shown (min, decreasing absorbance at 300 nm).

Table S1. Species observed in the nESI-FT-ICR mass spectra of solutions of complex **1** + (A) SubP and (B) K[3]-Bom, post-photoactivation.

(A) Complex **1**+Substance P

Species	Elemental composition	Modification to peptide observed
$[SubP+2H]^{2+}$	$C_{63}H_{98}N_{18}O_{13}SH_2$	N/A
$[SubP+H]^{+}$	$C_{63}H_{98}N_{18}O_{13}SH$	N/A
$[SubP+O+2H]^{2+}$	$C_{63}H_{98}N_{18}O_{13}SH_2O$	+O
$[SubP+\{Pt(py)_2\}+H]^{3+}$	$C_{63}H_{98}N_{18}O_{13}SPtC_{10}H_{10}N_2H$	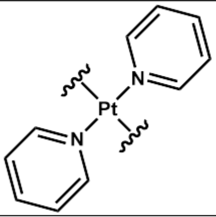
$[SubP+\{Pt(py)_2(N_3)\}+H]^{2+}$	$C_{63}H_{98}N_{18}O_{13}SPtC_{10}H_{10}N_5H$	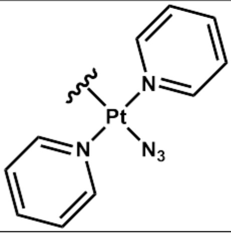
$[SubP+\{Pt(py)_2(OH)\}+H]^{2+}$	$C_{63}H_{98}N_{18}O_{13}SPtC_{10}H_{10}N_2OHH$	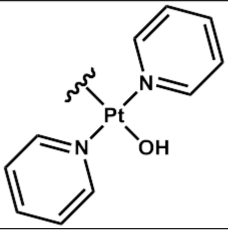
$[SubP+\{Pt(N_3)\}+H]^{2+}$	$C_{63}H_{98}N_{18}O_{13}SPtN_3H$	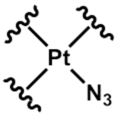
$[SubP+\{Pt(py)(OH)(N_3)\}+2H]^{2+}$	$C_{63}H_{98}N_{18}O_{13}SPtC_5H_5NOHN_3H_2$	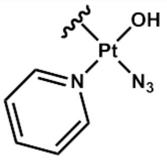
$[SubP+\{Pt(py)(OH)(N_3)\}+O+2H]^{2+}$	$C_{63}H_{98}N_{18}O_{13}SPtC_5H_5NOHN_3H_2O$	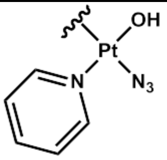 +O

(B) Complex **1**+K[3]-Bom

Species	Elemental composition	Modification to peptide observed
$[K^3\text{-}Bom+H]^{+}$	$C_{71}H_{110}N_{22}O_{18}SH$	N/A
$[K^3\text{-}Bom+2H]^{2+}$	$C_{71}H_{110}N_{22}O_{18}SH_2$	N/A
$[K^3\text{-}Bom+O+2H]^{2+}$	$C_{71}H_{110}N_{22}O_{18}SH_2O$	+O

$[K^3\text{-Bom}+2O+2H]^{2+}$	$C_{71}H_{110}N_{22}O_{18}SH_2O_2$	+2O
$[K^3\text{-Bom}+3O+2H]^{2+}$	$C_{71}H_{110}N_{22}O_{18}SH_2O_3$	+3O
$[K^3\text{-Bom}+\{Pt(py)_2\}]^{2+}$	$C_{71}H_{110}N_{22}O_{18}SHPtC_{10}H_{10}N_2$	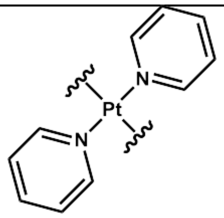
$[K^3\text{-Bom}+\{Pt(py)_2\}+H]^{3+}$	$C_{71}H_{110}N_{22}O_{18}SHPtC_{10}H_{10}N_2H$	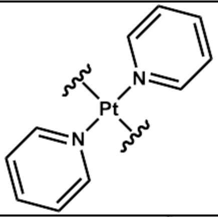
$[K^3\text{-Bom}+\{Pt(py)_2(OH)\}+H]^{2+}$	$C_{71}H_{110}N_{22}O_{18}SHPtC_{10}H_{10}N_2OHH$	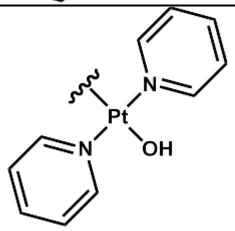
$[K^3\text{-Bom}+\{Pt(py)_2(N3)\}+H]^{2+}$	$C_{71}H_{110}N_{22}O_{18}SHPtC_{10}H_{10}N_2N_3H$	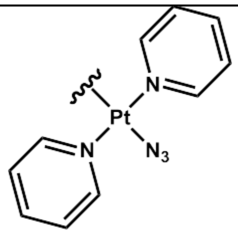
$[K^3\text{-Bom}+\{Pt(py)_2(N3)\}+O+H]^{2+}$	$C_{71}H_{110}N_{22}O_{18}SHPtC_{10}H_{10}N_2N_3OH$	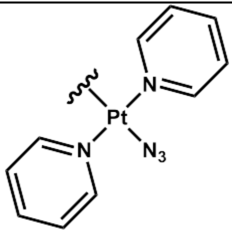 +O
$[K^3\text{-Bom}+\{Pt(py)_2(N3)\}+2O+H]^{2+}$	$C_{71}H_{110}N_{22}O_{18}SHPtC_{10}H_{10}N_2N_3O_2H$	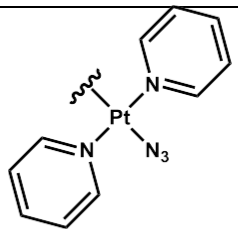 +2O
$[K^3\text{-Bom}+\{Pt(py)_2(N3)\}+3O+H]^{2+}$	$C_{71}H_{110}N_{22}O_{18}SHPtC_{10}H_{10}N_2N_3O_3H$	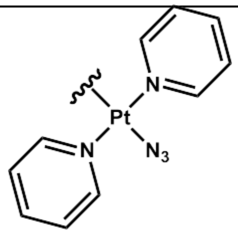 +3O

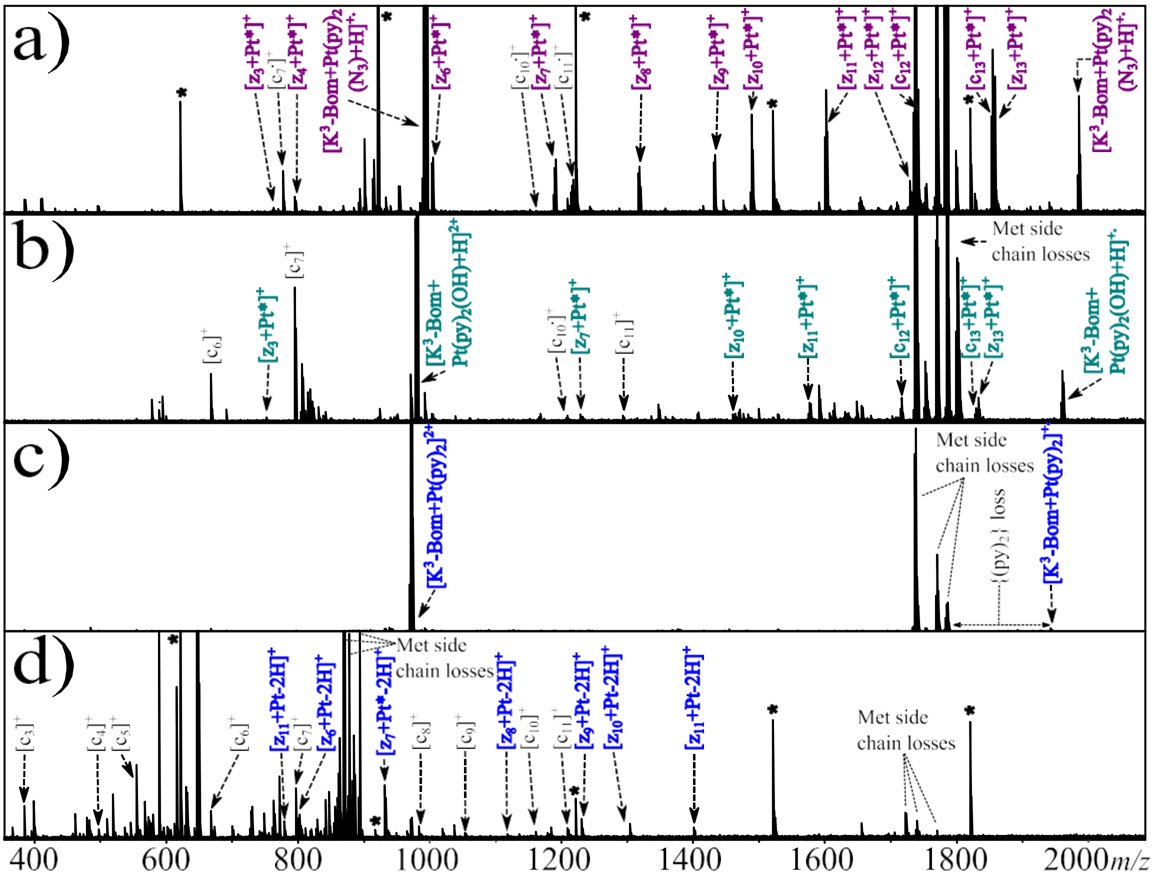

Figure S2. ECD MS/MS spectra of platinated K^3-Bom species individually isolated and dissociated using ECD within the FT-ICR MS a) [K^3-Bom+Pt(py)$_2$(N$_3$)+H]$^{2+}$ b) [K^3-Bom+Pt(py)$_2$(OH)+H]$^{2+}$ c) [K^3-Bom+Pt(py)$_2$]$^{2+}$ d) [K^3-Bom+Pt(py)$_2$+H]$^{3+}$. Coloured labels indicate modified fragments. Assignment Tables S7-9 lists the assignments for each species. Pt* indicates the platinum based modification associated with each species (see Figure 3, main text, also included in assignment Tables S7-9, above).

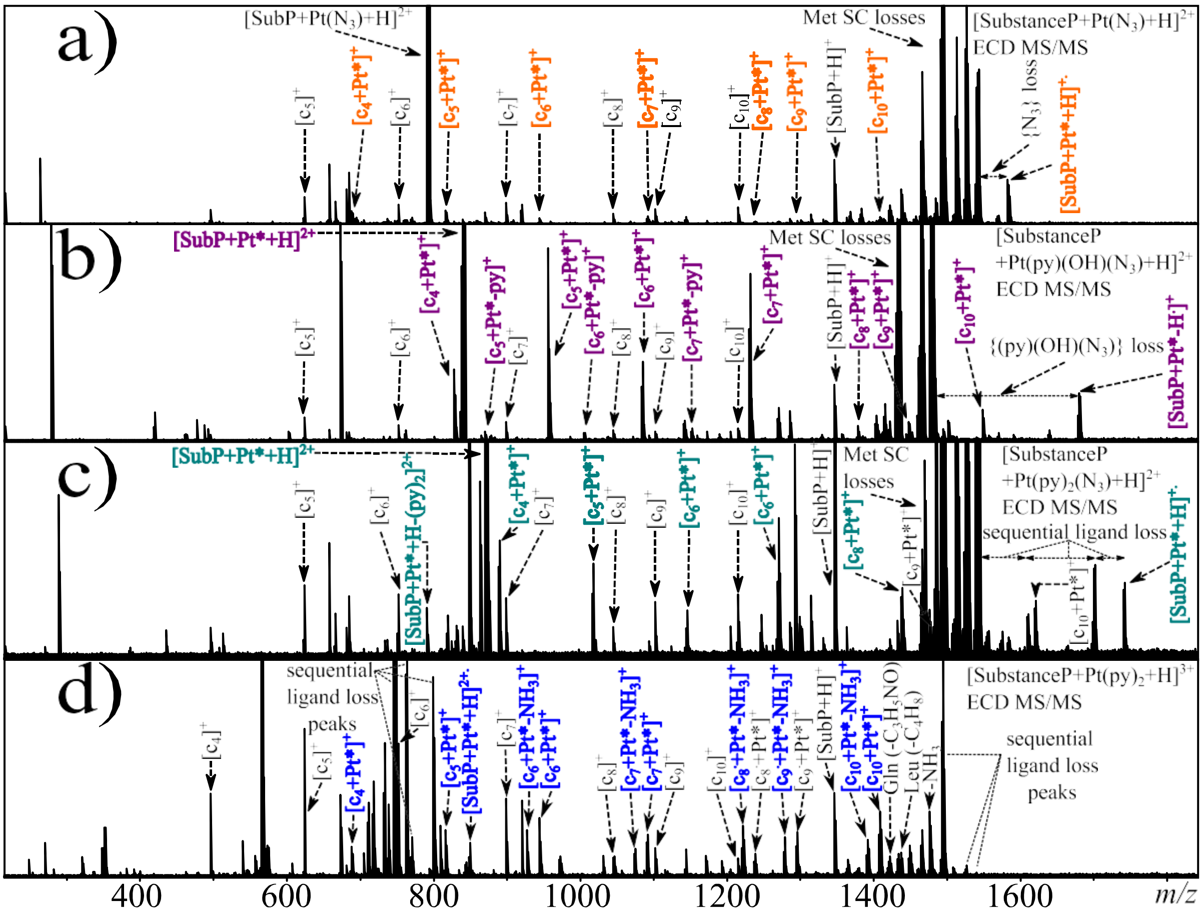

Figure S3. ECD MS/MS spectra of platinated Substance P species individually isolated and dissociated using ECD within the FT-ICR MS a) $[SubP+Pt(N_3)+H]^{2+}$ b) $[SubP+Pt(py)(OH)(N_3)+H]^{2+}$ c) $[SubP+Pt(py)_2(N_3)+H]^{2+}$ d) $[SubP+Pt(py)_2+H]^{3+}$. Coloured labels indicate modified fragments. Assignments for each species are in Tables S10-12. Pt* indicates the platinum-based modification associated with each species (see Figure 4, main text, also included in assignment Tables S10-12, above).

Tables S2-S7. Assignments for individual ECD MS/MS spectra of the isolated platinated peptide species. Marked species were used for internal calibration; in tables with no marked species, co-isolated calibrant ions were used instead of MS/MS fragments, as discussed in the Experimental section.

Table S2. ECD MS/MS assignments for the $[K^3\text{-Bom+Pt(py)}_2(N_3)\text{+H}]^{2+}$ species (calibrated using co-isolated calibrant ions):

Fragment	Exact mass	Observed mass	Error/ppm
$[c7^{\cdot}]^+$	796.418045	796.41805	0.01
$[c10^{\cdot}]^+$	1152.602875	1152.60367	0.69
$[z7\text{+ Pt(py)}_2(N_3)]^+$	1189.453306	1189.45339	0.07
$[c11^{\cdot}]^+$	1209.624335	1209.62400	-0.28
$[z8\text{+Pt(py)}_2(N_3)]^+$	1317.511886	1317.51124	-0.49
$[z9\text{+Pt(py)}_2(N_3)]^+$	1431.554816	1431.55358	-0.86
$[z10\text{+Pt(py)}_2(N_3)]^+$	1488.576276	1488.57688	0.41
$[z11\text{+Pt(py)}_2(N_3)]^+$	1601.660336	1601.66124	0.56
$[z12\text{+Pt(py)}_2(N_3)]^+$	1729.755296	1729.75603	0.42
$[c13\text{+Pt(py)}_2(N_3)]^+$	1853.823586	1853.82508	0.81
$[z13\text{+Pt(py)}_2(N_3)]^+$	1857.813876	1857.81385	-0.01
		Absolute average	-0.18
		Standard deviation	0.50

Table S3. ECD MS/MS assignments for the $[K^3\text{-Bom}+Pt(py)_2(OH)+H]^{2+}$ species (calibrated using co-isolated calibrant ions):

Fragment	Exact mass	Observed Mass	Error/ppm
$[c6]^+$	669.36729	669.36729	0.00
$[z3^{\cdot}+Pt(py)_2(OH)]^+$	752.248358	752.24755	-1.07
$[c7]^+$	797.42587	797.42614	0.34
$[c10^{\cdot}]^+$	1152.602875	1152.60293	0.05
$[z7^{\cdot}+Pt(py)_2(OH)]^+$	1165.454648	1165.45505	0.34
$[c11]^+$	1210.63216	1210.63215	-0.01
$[z10^{\cdot}+Pt(py)_2(OH)]^+$	1464.577618	1464.57681	-0.55
$[z11+Pt(py)_2(OH)]^+$	1576.653853	1576.65538	0.97
$[c12+Pt(py)_2(OH)]^+$	1715.733043	1715.73301	-0.02
$[c13+Pt(py)_2(OH)]^+$	1828.817103	1828.81907	1.08
$[z13+Pt(py)_2(OH)]^+$	1832.807393	1832.80709	-0.17
$[K^3\text{-Bom}+Pt(py)_2(OH)+H]^+$	1959.857593	1959.85800	0.21
		Absolute average	0.40
		Standard deviation	0.56

Table S4. ECD MS/MS assignments for the $[K^3\text{-Bom+Pt(py)}_2\text{+H}]^{3+}$ species:

Fragment	Exact mass	Observed Mass	Error/ppm
$[c3]^+$	385.2188	385.2188	0.00
$[c4]^+$	498.3029	498.3028	-0.26
$[c5]^+$	555.3244	555.3243	-0.05
$[c6]^+$	669.3673	669.3674	0.12
$[c7]^+$	797.4259	797.426	0.16
$[c8]^+$	983.5052	983.5048	-0.43
$[c9]^+$	1054.542	1054.542	-0.36
$[c10]^+$	1153.611	1153.611	0.33
$[c11]^+$	1210.632	1210.632	0.17
$[z6\text{-H}_2\text{-H+Pt}]^+$	802.2731	802.272	-1.40
$[z7\text{-H}_2\text{+Pt}]^+$	988.3524	988.3522	-0.21
$[z8\text{-H}_2\text{+Pt}]^+$	1116.411	1116.411	-0.29
$[z9\text{-H}_2\text{+Pt}]^+$	1230.454	1230.453	-0.43
$[z10\text{-H}_2\text{+Pt}]^+$	1287.475	1287.475	-0.63
$[z11\text{-H}_2\text{+Pt}]^+$	1400.559	1400.559	-0.65
$[z11\text{-2H+Pt(py)}_2]^{2+}$	779.8256	779.8247	-1.12
		Absolute average:	0.41
		Standard deviation:	0.46

Table S5. ECD MS/MS assignments for $[SubP+Pt(py)_2+H]^{3+}$ species:

Fragment	Exact mass	Observed mass	Error/ppm
$[c4^{\cdot}+Pt]^{+}$	688.28242	688.28242	0.00
$[c5^{\cdot}+Pt]^{+}$	816.34100	816.3412	0.24
$[c6^{\cdot}+Pt]^{+}$	944.39958	944.39917	-0.43
$[c7^{\cdot}+Pt]^{+}$	1091.46799	1091.46803	0.04
$[c8^{\cdot}+Pt]^{+}$	1238.5364	1238.53627	-0.10
$[c9^{\cdot}+Pt]^{+}$	1295.55786	1295.55822	0.28
$[c10^{\cdot}+Pt]^{+}$	1408.64192	1408.64168	-0.17
$[c5^{\cdot}+Pt-NH_3]^{+}$	799.314455	799.31421	-0.31
$[c6^{\cdot}+Pt-NH_3]^{+}$	927.373035	927.37283	-0.22
$[c7^{\cdot}+Pt-NH_3]^{+}$	1074.441445	1074.44128	-0.15
$[c8^{\cdot}+Pt-NH_3]^{+}$	1221.509855	1221.50967	-0.15
$[c9^{\cdot}+Pt-NH_3]^{+}$	1278.531315	1278.53071	-0.47
$[c10^{\cdot}+Pt-NH_3]^{+}$	1391.615375	1391.61515	-0.16
$[SubP+Pt]^{+\cdot}-H^{\cdot}$	1539.681866	1539.68187	0.00
$[SubP+Pt]^{+\cdot}-H^{\cdot}-Me$	1524.658939	1524.65993	0.65
$[SubP+Pt]^{+\cdot}-MeS$	1493.694694	1493.69432	-0.25
$[SubP+Pt]^{+\cdot}-MeS-NH_3$	1476.668145	1476.66816	0.01
$[SubP+Pt]^{+\cdot}$-MeS-Leu C_4H_8	1437.632094	1437.63193	-0.11
$[SubP+Pt]^{+\cdot}$-MeS-Glu C_3H_5NO	1422.65758	1422.65785	0.19
		Absolute average	0.21
		Standard deviation	0.26

Table S6. ECD MS/MS assignments for $[SubP+Pt(py)_2(N_3) +H]^{2+}$ species:

Fragment	Exact mass	Observed mass	Error/ppm
$[c4+Pt(py)_2(N_3)]^+$	889.383866	889.38374	-0.14
$[c5+Pt(py)_2(N_3)]^+$	1017.442446	1017.44264	0.19
$[c6+Pt(py)_2(N_3)]^+$	1145.501026	1145.50134	0.27
$[c7+Pt(py)_2(N_3)]^+$	1292.569436	1292.57007	0.49
$[z9-H^{\cdot}+Pt(py)_2(N_3)]^+$	1470.603986	1470.60669	1.84
$[c10+Pt(py)_2(N_3)]^+$	1609.743366	1609.74471	0.83
$[SubP+Pt(py)_2(N_3)+H]^{+\cdot}$	1740.783856	1740.78386	0.00
$[SubP+Pt]^+$	1540.690813	1540.69154	0.47
$[SubP+Pt-Me]^+$	1525.667338	1525.66709	-0.16
$[SubP+Pt-S]^+$	1493.695268	1493.69489	-0.25
		Absolute average	0.47
		Standard deviation	0.59

Table S7. ECD MS/MS assignments for $[SubP+Pt(N_3)+H]^{2+}$ species:

Fragment	Exact mass	Observed mass	Error/ppm
$[c4^{\cdot}+Pt]^{+}$	688.28242	688.28242	0.00
$[c5^{\cdot}+Pt]^{+}$	816.341	816.34078	-0.27
$[c6^{\cdot}+Pt]^{+}$	944.39958	944.39958	0.00
$[c7^{\cdot}+Pt]^{+}$	1091.46799	1091.46827	0.26
$[c8^{\cdot}+Pt]^{+}$	1238.5364	1238.53767	1.03
$[c9^{\cdot}+Pt]^{+}$	1295.55786	1295.55875	0.69
$[c10^{\cdot}+Pt]^{+}$	1408.64192	1408.64181	-0.08
$[SubP+Pt(N_3)+H]^{+\cdot}$	1582.69837	1582.69848	0.07
		Absolute average	0.30
		Standard deviation	0.41

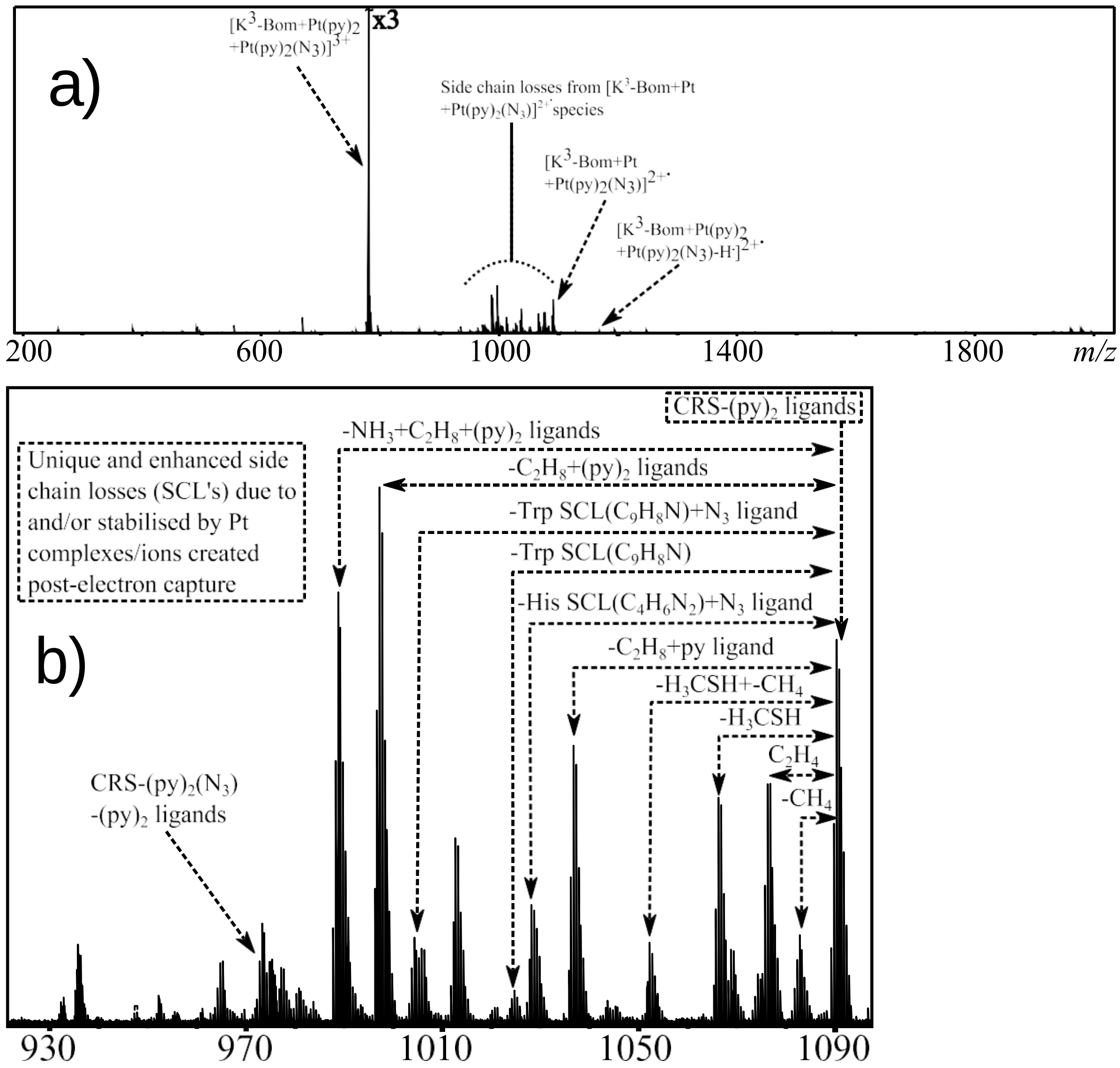

Figure S4. (a) ECD MS/MS spectra of di-platinated K^3-bom species, and (b) expansion of side chain loss (SCL) region resulting from electron capture at a platinum centre, causing ligand loss (to create [K^3-Bom+Pt+Pt(py)$_2$(N$_3$)]$^{2+\cdot}$ from the [K^3-Bom+Pt(py)$_2$+Pt(py)$_2$(N$_3$)]$^{2+\cdot}$ species) along with unique side chain losses. Unfortunately no sequence informative fragments were observed, highlighting the need for an additional proton to allow standard dissociation.

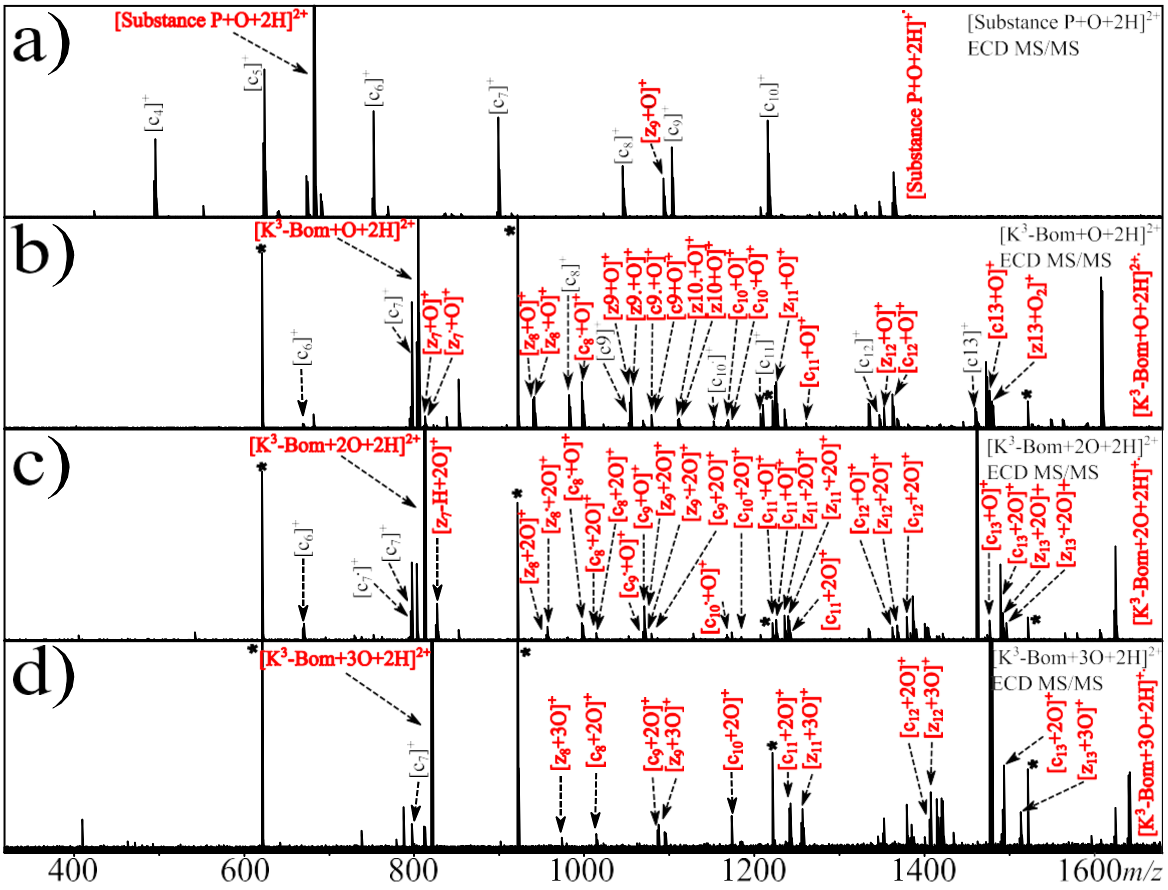

Figure S5. ECD MS/MS spectra of oxidised peptides individually isolated and dissociated using ECD within the FT-ICR MS. a) Substance P+O. b) K^3-Bom+O. C) K^3-Bom+2O. d) K^3-Bom+3O. Red labels indicate modified fragments. Assignments for each species are in Tables S3-S6 for spectra S2a-d respectively.

Tables S8-S11. Aassignments for individual ECD MS/MS spectra of the isolated oxidised peptide species. **S8**: Substance P+O; **S9**: K^{3}-Bom+O; **S10**: K^{3}-Bom+2O; **S11**: K^{3}-Bom+3O. Marked species were used for internal calibration; in tables with no marked species, co-isolated calibrant ions were used instead of MS/MS fragments, as discussed in the Experimental section.

Table S8. ECD MS/MS assignments for SubP+O species.

Fragment	Exact mass	Observed mass	Error/ppm
$[c2]^{+}$	271.18769	271.1877	0.04
$[c4]^{+}$	496.33541	496.33543	0.04
$[c5]^{+}$	624.39399	624.394	0.02
$[c6]^{+}$	752.45257	752.45257	0.00
$[c7]^{+}$	899.52098	899.52094	-0.04
$[c8]^{+}$	1046.58939	1046.58935	-0.04
$[c9]^{+}$	1103.61085	1103.61064	-0.19
$[c10]^{+}$	1216.69491	1216.69471	-0.16
$[SubP+O+2H]^{+\cdot}$	1364.738145	1364.73817	0.02
$[z9]^{+}$	1094.557725	1094.55765	-0.07
		Absolute average	0.06
		Standard deviation	0.08

Table S9. ECD MS/MS assignments for K^3-Bom+O species.

Fragment	Exact mass	Measured mass	Error/ppm
$[c6]^{+}$	669.36729	669.36794	0.97
$[c7]^{+}$	797.42587	797.42638	0.64
$[c9]^{+}$	1070.537205	1070.53756	0.33
$[c10]^{+}$	1169.605615	1169.6061	0.41
$[c11]^{+}$	1226.627075	1226.6277	0.51
$[c12]^{+}$	1363.685985	1363.68616	0.13
$[c13]^{+}$	1476.770045	1476.77013	0.06
$[K^3\text{-Bom+O+2H-H}]^{+\cdot}$	1607.810535	1607.80981	-0.45
$[c8^{\cdot}]^{+}$	998.49227	998.4925	0.23
$[c9^{\cdot}]^{+}$	1069.52938	1069.52972	0.32
$[c10^{\cdot}]^{+}$	1168.59779	1168.59847	0.58
$[z7]^{+}$	812.399765	812.4003	0.66
$[z8]^{+}$	940.458345	940.45818	-0.18
$[z9]^{+}$	1054.501275	1054.50101	-0.25
$[z10]^{+}$	1111.522735	1111.52226	-0.43
$[z11]^{+}$	1224.606795	1224.60649	-0.25
$[z12]^{+}$	1352.701755	1352.70124	-0.38
$[z13]^{+}$	1480.760335	1480.75951	-0.56
$[z7^{\cdot}]^{+}$	813.40759	813.40772	0.16
$[z8^{\cdot}]^{+}$	941.46617	941.46654	0.39
$[z9^{\cdot}]^{+}$	1055.5091	1055.50884	-0.25
$[z10^{\cdot}]^{+}$	1112.53056	1112.53004	-0.47
		Absolute average	0.39
		standard deviation	0.43

Table S10. ECD MS/MS assignments for K^3-Bom+2O species.

Fragment	Exact mass	Observed Mass	Error/ppm
$[c6]^+$	669.36729	669.36795	0.99
$[c7.\ ]^+$	796.41805	796.41856	0.65
$[c7]^+$	797.42587	797.42640	0.66
$[z7\text{-}H+2O]^+$	827.38740	827.38688	-0.63
$[z8+2O]^+$	956.45326	956.45340	0.15
$[z8.+2O]^+$	957.46109	957.46118	0.10
$[c8.+O]^+$	998.49227	998.49253	0.26
$[c8.+2O]^+$	1014.48719	1014.48791	0.71
$[c8+2O]^+$	1015.49501	1015.49604	1.01
$[c9.+O]^+$	1069.52938	1069.53030	0.86
$[c9+O]^+$	1070.53721	1070.53746	0.24
$[z9+2O]^+$	1070.49619	1070.49576	-0.40
$[z9^{\cdot}+2O]^+$	1071.50402	1071.50399	-0.02
$[c9+2O]^+$	1086.53212	1086.53442	2.12
$[c10^{\cdot}+O]^+$	1168.59779	1168.59867	0.75
$[c10+2O]^+$	1185.60053	1185.60047	-0.05
$[c11^{\cdot}+O]^+$	1225.61925	1225.61937	0.10
$[c11+O]^+$	1226.62708	1226.62731	0.19
$[z11+2O]^+$	1240.60171	1240.60117	-0.44
$[z11^{\cdot}+2O]^+$	1241.60954	1241.60878	-0.61
$[c11+2O]^+$	1242.62199	1242.62276	0.62
$[c12+O]^+$	1363.68599	1363.68585	-0.10
$[z12+2O]^+$	1368.69667	1368.69605	-0.45
$[c12+2O]^+$	1379.68090	1379.68053	-0.27
$[c13+O]^+$	1476.77005	1476.77044	0.27
$[c13+2O]^+$	1492.76496	1492.76449	-0.31
$[z13+2O]^+$	1496.75525	1496.75359	-1.11
$[z13^{\cdot}+2O]^+$	1497.76308	1497.76135	-1.15
		Absolute average	0.54
		Standard deviation	0.69

Table S11. ECD MS/MS assignments for K^3-Bom+3O species.

Fragment	Exact Mass	Observed Mass	Error/ppm
$[c7]^{+}$	797.42587	797.42619	0.40
$[c9+2O]^{+}$	1086.53212	1086.53357	1.33
$[c11+2O]^{+}$	1242.62199	1242.6212	-0.64
$[c12+2O]^{+}$	1379.6809	1379.68204	0.83
$[c13+2O]^{+}$	1492.76496	1492.76538	0.28
$[K^3\text{-Bom}+3O+2H-H]^{+\cdot}$	1639.800365	1639.80098	0.38
$[c8^{\cdot}+2O]^{+}$	1014.487185	1014.48766	0.47
$[c10^{\cdot}+2O]^{+}$	1184.592705	1184.59291	0.17
$[c13^{\cdot}+2O]^{+}$	1491.757135	1491.75668	-0.31
$[z11+3O]^{+}$	1256.596625	1256.5958	-0.66
$[z12+3O]^{+}$	1384.691585	1384.69032	-0.91
$[z13+3O]^{+}$	1512.750165	1512.74992	-0.16
$[z8^{\cdot}+3O]^{+}$	973.45600	973.45558	-0.43
$[z9^{\cdot}+3O]^{+}$	1087.49893	1087.49961	0.63
		Absolute average	0.54
		Standard deviation	0.62

Table 12. EPR data for irradiation with blue visible light of (A) complex **1** in the presence of the spin-trap DEPMPO, (B) +SubstanceP, (C) +[Lys]^3-Bombesin. Included are previously published EPR parameters for the platinum[1] or oxidised DEPMPO[2,3] species observed.

(A) Complex 1+DEPMPO (DEPMPO-N_3)

	g	a^N_{NO}	a^P	$a^H_β$	$a^N_α$
**Experimental**	2.012	13.89	46.16	13	2.79
**Lit**[1]	-	13.93	46.05	12.39	2.80

(B) Complex 1+Substance P+DEPMPO (DEPMPO-N_3)

	g	a^N_{NO}	a^P	$a^H_β$	$a^N_α$
**Experimental**	2.012	13.89	46.16	13	2.79
**Lit**[1]	-	13.93	46.05	12.39	2.80

(C) Complex 1+[Lys]^3-Bombesin+DEPMPO (DEPMPOX)

	g	a^N_{NO}	a^P	$a^H_β$
**Experimental**	2.012	7.08	38.75	3.76
**Lit**[2,3]	-	7.8/7.14	41.2/38.69	4.1/3.69

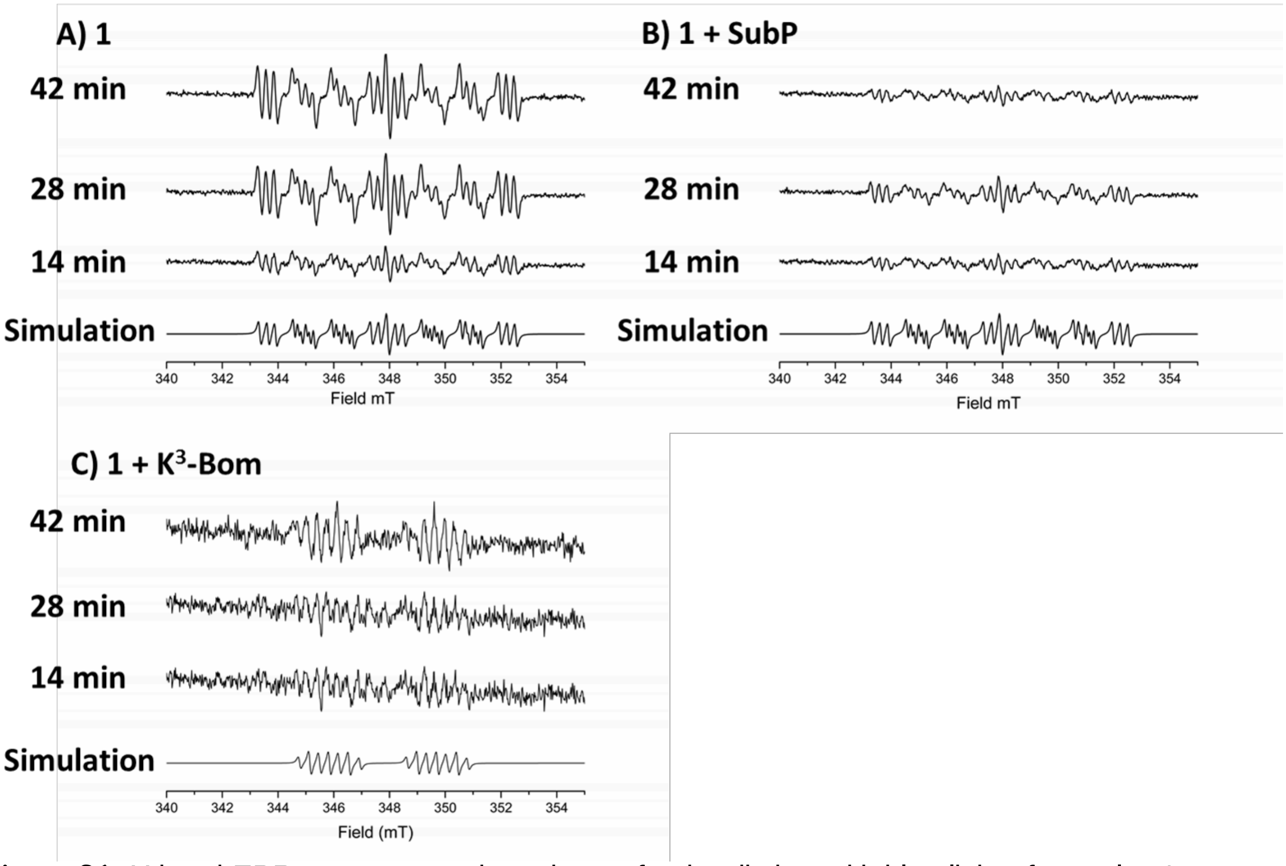

Figure S6. X-band EPR spectra at various times after irradiation with blue light of complex 1 + DEMPO + (a) no peptide, (b) SubP (c) K^3-Bom. Simulations show spectra for adducts (a) DEMPO-N_3, (b) DEMPO-N_3, (c) DEMPOX.

Table 13. Relative intensities of peaks for assigned species in the FTMS of irradiated complex **1**+ (A) Substance P, and (B) K^3-Bom reaction mixtures, with and without added tryptophan.

(A) 1+Sub P

Species	Relative Intensity/%	
	-Trp	+Trp[a]
$[SubP+2H+O]^{2+}$	11.02	7.82
$[SubP+Pt(py)_2]^{2+}$	0.81	1.24
$[SubP+Pt(py)(OH)(N_3)+H]^{2+}$	4.97	3.94
$[SubP+Pt(py)(OH)(N_3)+H+O]^{2+}$	0.81	0.04
$[SubP+Pt(N)_3+H]^{2+}$	2.46	2.54
$[SubP+Pt(py)_2(N)_3+H]^{2+}$	4.28	3.01

[a] Tryptophan was added to 1/8th the concentration of **1** (end Trp concentration 31.25µM, 3.1 nMoles Trp added).

(B) 1+K^3-Bom

Species	Relative Intensity/%	
	-Trp	+Trp
$[K^3\text{-Bom}+2H+O]^{2+}$	3.04	4.04
$[K^3\text{-Bom}+2H+2O]^{2+}$	11.97	7.19
$[K^3\text{-Bom}+2H+3O]^{2+}$	15.7	16.13
$[K^3\text{-Bom}+Pt(py)_2(N_3)+H]^{2+}$	24.40	7.20
$[K^3\text{-Bom}+Pt(py)_2(N_3)+H+O]^{2+}$	0.50	0.07
$[K^3\text{-Bom}+Pt(py)_2(N_3)+2H+O]^{3+}$	0.03	0.01
$[K^3\text{-Bom}+Pt(py)_2(OH)+H]^{2+}$	2.28	0.05
$[K^3\text{-Bom}+Pt(py)_2]^{2+}$	15.34	0.80
$[K^3\text{-Bom}+Pt(py)_2+H]^{3+}$	28.87	1.23
$[K^3\text{-Bom}+Pt(py)_2+H+O]^{3+}$	0.47	0.01
$[K^3\text{-Bom}+Pt(py)_2+H+2O]^{3+}$	1.90	0.05
$[K^3\text{-Bom}+Pt(py)_2+Py(py)_2(N_3)]^{3+}$	0.86	0.02

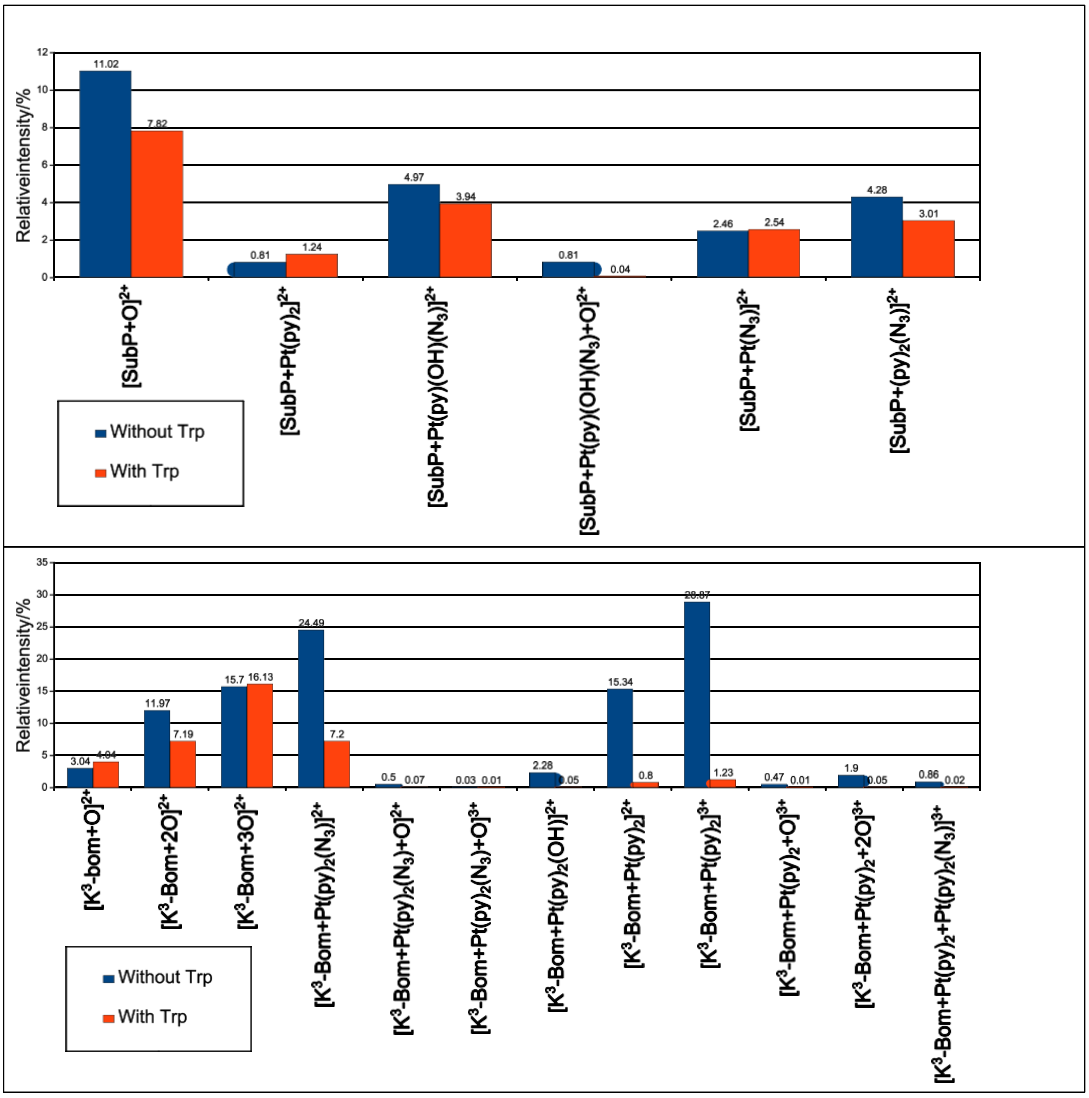

Figure S7. Barcharts showing the relative intensity changes for MS peaks for the products of reactions between **1**+peptide and **1**+peptide+Trp for both **SubP** (top) and **K^3-Bom** (bottom). Intensities are listed as a percentage of the base peak observed in the spectrum. Tryptophan added was 1/8th the concentration of **1** (final concentration 31.25µM, 3.1 nmol Trp added).

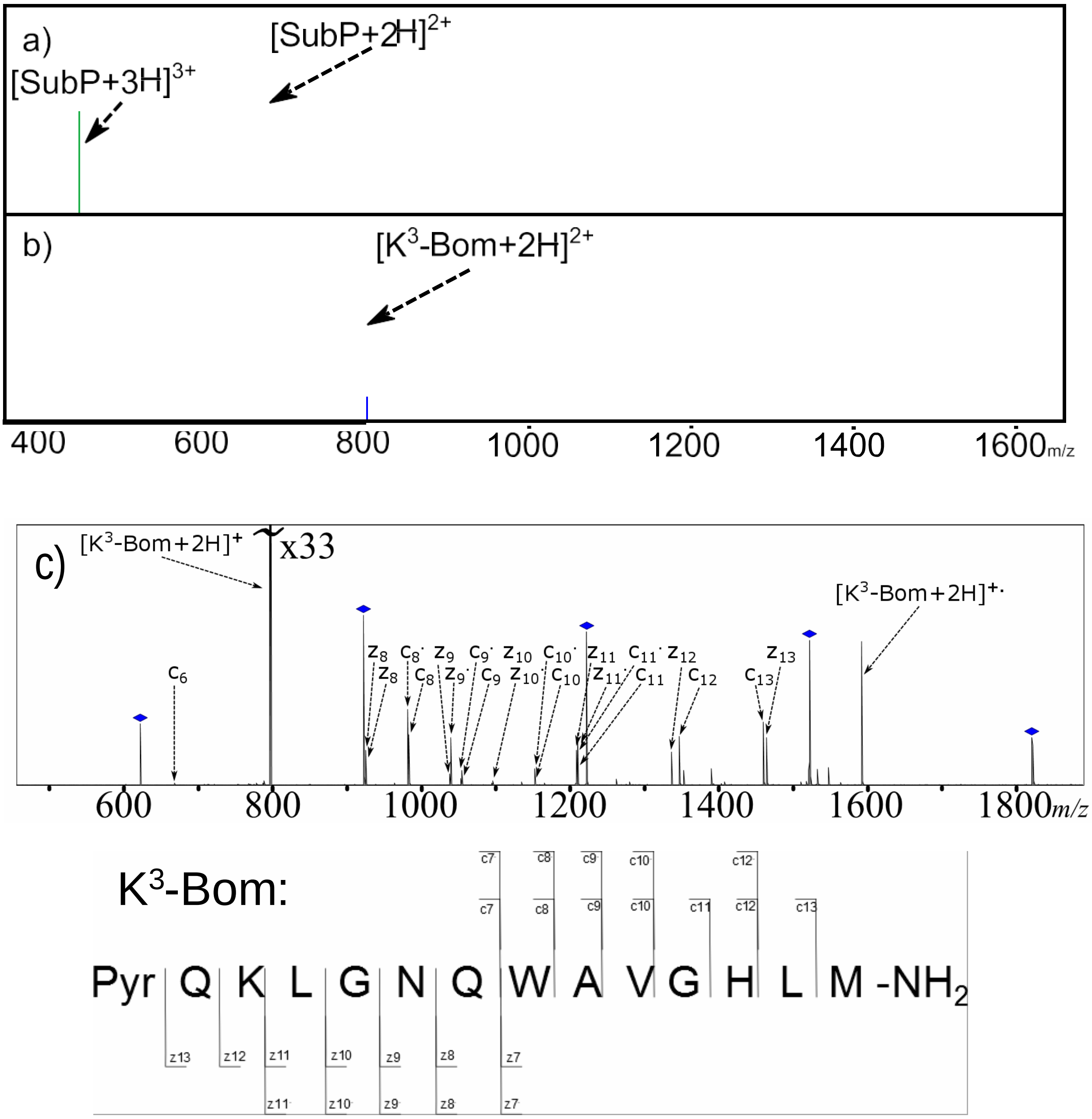

Figure S8. (a) nESI-FT-ICR MS spectrum of (a) Substance P and (b) K[3]-Bom; (c) ECD MS/MS spectrum of K[3]-Bom; peptide was co-isolated with 5 calibrant ions to improve calibration and compare the use of peptide MS/MS fragments and unrelated ions when internally calibrating MS/MS spectra.

Table S14. Peptide fragment assignments and associated mass errors for the ECD MS/MS spectra of the $[K^3\text{-Bom}+2H]^{2+}$ species with and without the use of co-isolated calibrant ions.

Using co-isolated HP mix calibrant ions:			
Assignment	Theoretical m/z	Observed m/z	Error/ ppm
c6	669.36729	669.3678	0.76
z7	796.40485	796.40441	-0.55
c7	797.42587	797.42642	0.69
z8	924.46343	924.46324	-0.21
z8.	925.471255	925.4709	-0.38
c8.	982.497355	982.49757	0.22
c8	983.50518	983.50543	0.25
z9	1038.50636	1038.50604	-0.31
z9.	1039.514185	1039.51385	-0.32
c9.	1053.534465	1053.53491	0.42
c9	1054.54229	1054.54262	0.31
z10	1095.52782	1095.52701	-0.74
z10.	1096.535645	1096.5349	-0.68
c10.	1152.602875	1152.60309	0.19
c10	1153.6107	1153.61108	0.33
z11	1208.61188	1208.61158	-0.25
z11.	1209.619705	1209.61878	-0.76
c11.	1209.624335	1209.62488	0.45
c11	1210.63216	1210.63259	0.36
z12	1336.70684	1336.70647	-0.28
c12	1347.69107	1347.69166	0.44
c13	1460.77513	1460.77538	0.17
z13	1464.76542	1464.7654	-0.01
CRS-H	1591.81562	1591.81624	0.39
	Average error		0.40
	Standard deviation		0.44

Using K^3-Bom fragment ions:			
Assignment	Theoretical m/z	Observed m/z	Error/ ppm
c6	669.36729	669.36729	0.00
z7	796.40485	796.40386	-1.24
c7	797.42587	797.42587	0.00
z8	924.46343	924.4627	-0.79
z8.	925.471255	925.47035	-0.98
c8.	982.497355	982.49703	-0.33
c8	983.50518	983.50489	-0.29
z9	1038.50636	1038.50552	-0.81
z9.	1039.514185	1039.51333	-0.82
c9.	1053.534465	1053.53439	-0.07
c9	1054.54229	1054.54211	-0.17
z10	1095.52782	1095.52652	-1.19
z10.	1096.535645	1096.5344	-1.14
c10.	1152.602875	1152.60262	-0.22
c10	1153.6107	1153.61061	-0.08
z11	1208.61188	1208.61114	-0.61
z11.	1209.619705	1209.61834	-1.13
c11.	1209.624335	1209.62444	0.09
c11	1210.63216	1210.63215	-0.01
z12	1336.70684	1336.70612	-0.54
c12	1347.69107	1347.69133	0.19
c13	1460.77513	1460.77515	0.01
z13	1464.76542	1464.7652	-0.15
CRS-H	1591.81562	1591.81617	0.35
	Average error		0.47
	Standard deviation		0.51

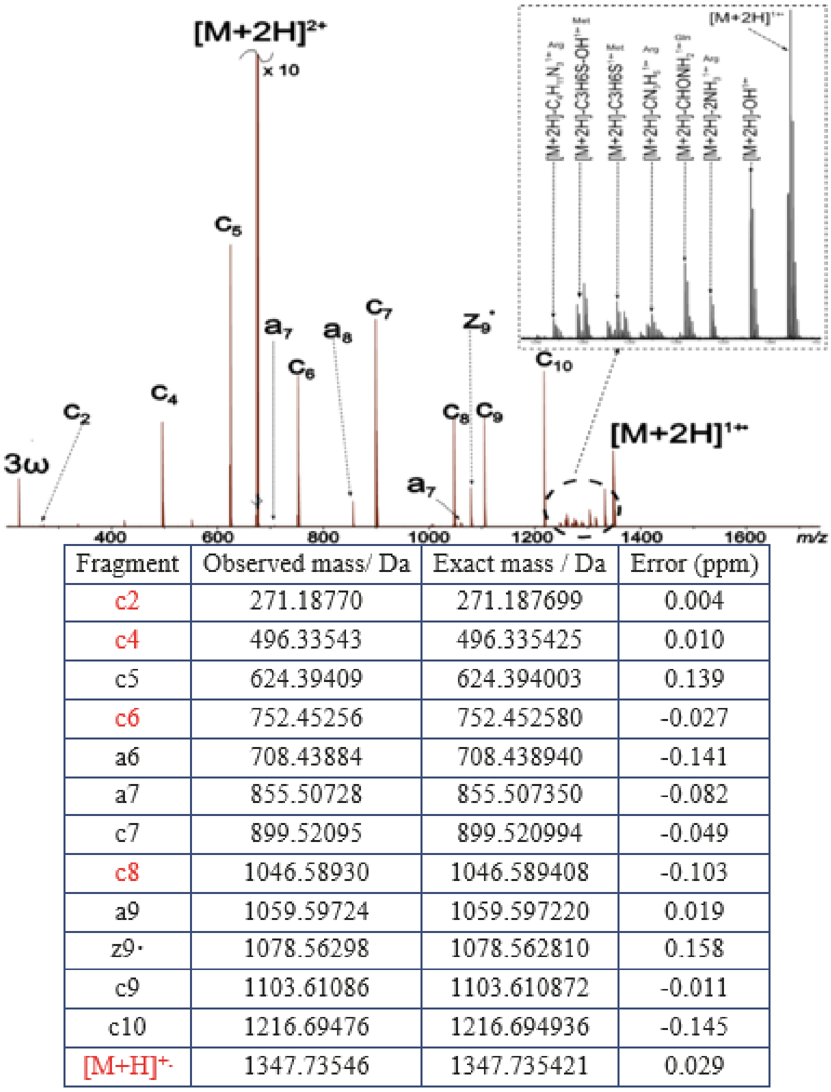

Fragment	Observed mass/ Da	Exact mass / Da	Error (ppm)
c2	271.18770	271.187699	0.004
c4	496.33543	496.335425	0.010
c5	624.39409	624.394003	0.139
c6	752.45256	752.452580	-0.027
a6	708.43884	708.438940	-0.141
a7	855.50728	855.507350	-0.082
c7	899.52095	899.520994	-0.049
c8	1046.58930	1046.589408	-0.103
a9	1059.59724	1059.597220	0.019
z9•	1078.56298	1078.562810	0.158
c9	1103.61086	1103.610872	-0.011
c10	1216.69476	1216.694936	-0.145
[M+H]+•	1347.73546	1347.735421	0.029

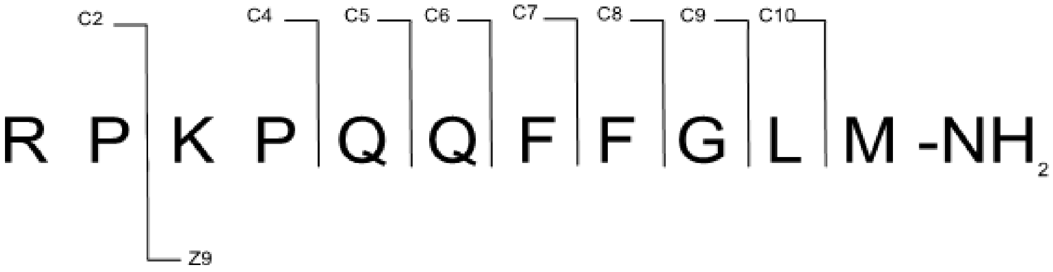

Figure S9. ECD MS/MS of unmodified Substance P (top), a list of assignments (middle), and corresponding fragmentation map (bottom)

Effects of platinum on MS/MS fragmentation

The presence of the Pt^{II} centre caused a dramatic effect on both electron based and collision based MS/MS experiments (ECD and CAD). The charged Pt centre altered the ECD MS/MS fragmentation pattern by providing a fixed charge at the point of interaction (e.g. His^{11} of K^3-Bom), but also enhanced side chain losses of amino acid groups present. Electron capture at the Pt^{II} centre/electron transfer of previously captured electrons on the peptide also caused additional ligand loss from the bound complex and additional fragmentations from amino acid residues. CAD MS/MS of platinum complex-containing species caused gas-phase dissociation of the platinum-bound ligands, even at low energies (<5-7V), creating a reactive Pt^{II} centre, which quickly cyclised with available peptide groups and produced uninformative fragmentation spectra.

The lack of backbone fragmentation is attributable to both the positive charge on Pt^{II}, without additional protons along the poly(amino acid) backbone, causing the normal electron capture dissociation mechanism to be disrupted/impeded. Electron capture by Pt can dominate, causing release of bound ligands and side chain losses from nearby amino acids.

Effect of Pt on electron dissociations

The ECD MS/MS spectrum of each Pt adduct showed a series of high intensity, singly-charged peaks close to the charge-reduced species (Fig. S3a-d and S4a-d). These peaks did not match any calculated usual c or z ions, nor any side chain loss commonly observed during ECD,[1] but the highest *m/z* peak did correspond to the mass of the peptide+Pt (e.g. $[\text{Substance P+Pt}]^+$ for the $[\text{Substance P+Pt(py)}_2(\text{N}_3)\text{+H}]^{2+}$ species), indicating an ECD-induced loss of ligands from Pt. The peak spacings were 15.02374m/z and 31.97186m/z. This corresponds to two separate losses of a terminal methyl group $^{\cdot}CH_3$ (-0.16 ppm) and loss of $^{\cdot}CH_3$-SH (48.003920 m/z, -0.25 ppm) (SI Fig. S7). Considering the sequences of Substance P (RPKPQQFFGLM-NH_2) and K^3-Bom (pEQKLGNQWAVGHLM-NH_2) the only sulphur atom present is within the thioether side chain of methionine. The observed side chain loss differs from the usual side chain losses commonly observed for methionine which are C_3H_6S or C_2H_4S,[1] suggesting that the Pt centre has caused the unusual fragmentation of the peptide under ECD.[2] For this to occur, Pt would have to be in close proximity to the methionine residue, as seen in previous work by Li *et. al.,*[3] yet all current data points clearly to the binding of Pt to the histidine (His^{11}) and lysine (Lys^3) residues, depending on the amino acid sequence. Methionine sulphur is known to be a strong ligand for Pt(II),[4,5] and the side chain losses here indicate a localised interaction. As a result this localised reaction must occur after the electron capture-induced loss of the Pt-bound ligands. The methionine side chain can then coordinate to vacant sites on the Pt centre and account for the side chain losses observed. A proposed mechanism for the interaction of a methionine residue and the subsequent loss of the terminal methyl and CH_3-SH species from the peptide is shown in Scheme S1. This scheme correlates with the side chain losses observed during this study, with the predicted interaction with methionine found in several reported Pt peptide MS/MS studies,[5,6,7] Pt- centred capture of an electron during ECD,[3] and commonly observed in Pt-ligand binding studies in solution.[8,9]

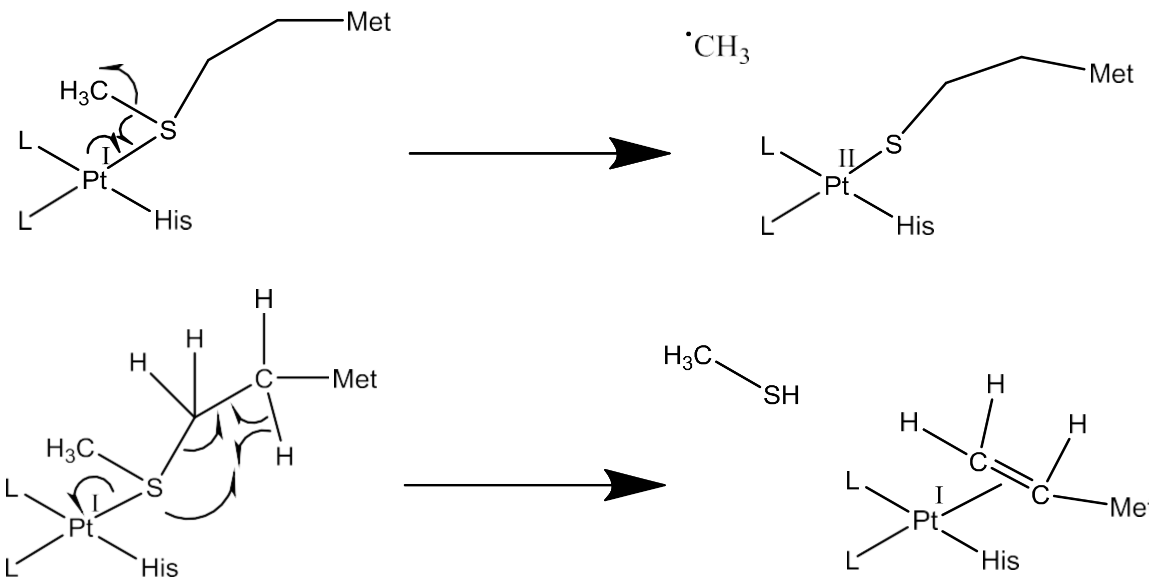

Scheme S1: Platinum-centred side chain losses from methionine commonly observed during ECD MS/MS studies of platinated peptides.

Scheme S2 shows a proposed mechanism for the loss of the platinum complex from peptides during electron capture, often observed for singly bound Pt modifications in this study (single binding site on peptide).

ECD MS/MS of the diplatinated K^3-Bom species produced a similar array of side chain losses as observed in mono-platinated species, however a more extensive number and variety of unusual sidechain losses and fragmentation channels were observed (SI Figure SF10). Unfortunately as observed in the ECD MS/MS of the $[K^3\text{-Bom+Pt(py)}_2]^{2+}$ species, without an additional proton, ECD MS/MS was unable to provide enough sequence-informative fragments to assign binding positions of the two platinum complexes, though based on the data observed for the previous modifications, tentatively these modifications would be expected to be present on the His^{12} and Met^{14} residues (for $\{Pt(py)_2\}$) and the Lys^3 (for $\{Pt(py)_2(N_3)\}^+$).

Effect of Pt on slow heating dissociations

It should be noted that although more commonly used Collisionally Activated Dissociation (CAD) and Infra-Red Multi Photon Dissociation (IRMPD) MS/MS techniques were attempted on the observed platinum-peptide adducts, these ergodic/"slow heating" fragmentation methods break bonds in the lowest energy pathways in a molecule.[10,11] For peptides, these usually involve the amide bonds along the backbone. However, CAD and IRMPD on the Pt adducts, resulted in a series of peaks corresponding to the loss of each platinum-bound ligand present, loss of the platinum modification (unmodified peptide), and a series of unassignable Pt and non-Pt containing peaks due to sequence scrambling commonly observed during the study of peptides and (gas-phase) labile post translational modifications such as metallation.[12,13] In contrast, ligand loss was observed due electron capture at the metal centre during ECD MS/MS of the Pt adducts studied here. Electrons

could also be captured at points along the backbone according to the accepted ECD MS/MS mechanism,[14] allowing normal ECD fragmentation and production of interpretable c/z ions.

Although the Pt complex showed the potential to bind to multiple groups present on the peptides studied; no crosslinking was observed, despite the low drug-to-peptide mol ratio (0.5:1) which might have promoted this possibility. The lack of crosslinking could be due to the short irradiation (reaction) times used (compared to the ~24 hour times used for cisplatin crosslinking studies)[5] and the low concentrations of reagents.[6] Square-planar Pt(II) has previously shown to act as an effective crosslinking agent, even for proteins such as calmodulin.[6]

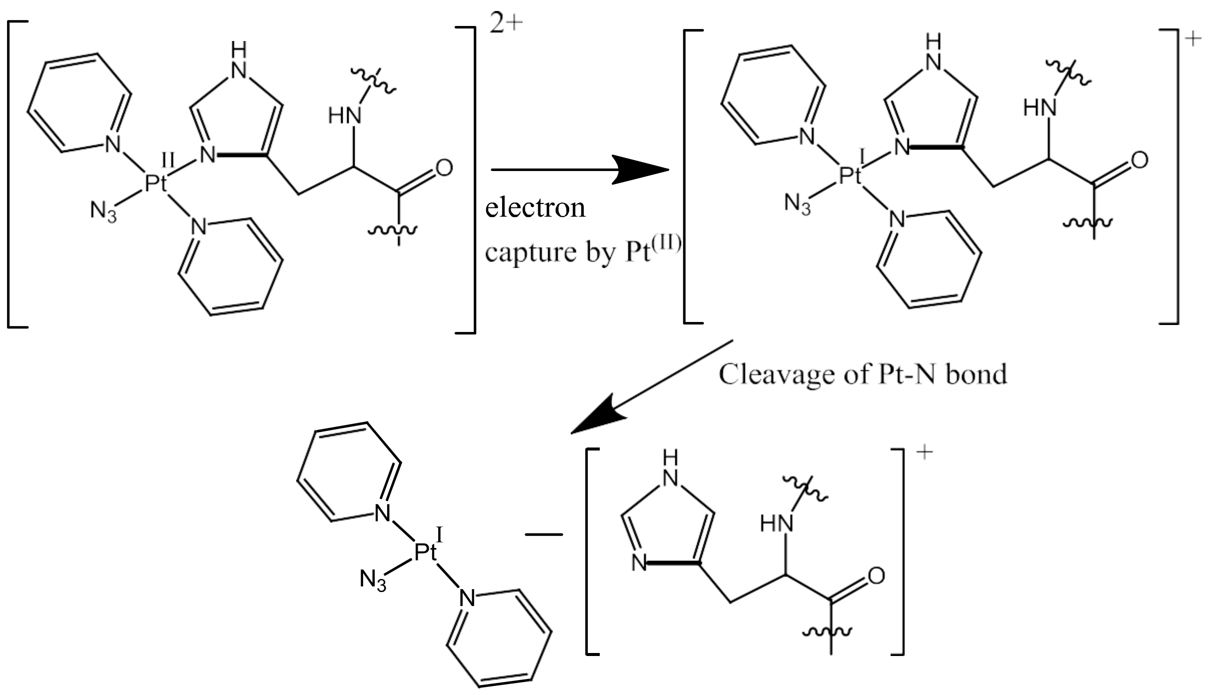

Scheme S2. Proposed mechanism for the loss of the Pt(II) modification from the peptide during ECD; ligands shown vary with the nature of the Pt adduct.

References

1 H. J. Cooper, R. R. Hudgins, K. Håkansson and A. G. Marshall, *J. Am. Soc. Mass Spectrom.*, 2002, **13**, 241–9.

2 H. Li, J. R. Snelling, M. P. Barrow, J. H. Scrivens, P. J. Sadler and P. B. O'Connor, *J. Am. Soc. Mass Spectrom.*, 2014, **25**, 1217–1227.

3 H. Li, T. Lin, S. L. Van Orden, Y. Zhao, M. P. Barrow, A. M. Pizarro, Y. Qi, P. J. Sadler and P. B. O. Connor, *Anal. Chem.*, 2011, **83**, 9507–9515.

4 H. Li, T. Lin, S. L. Van Orden, Y. Zhao, M. P. Barrow, A. M. Pizarro, Y. Qi, P. J. Sadler and P. B. O'Connor, *Anal. Chem.*, 2011, 9507–9515.

5 H. Li, Y. Zhao, H. I. A. Phillips, Y. Qi, T.-Y. Lin, P. J. Sadler and P. B. O'Connor, *Anal. Chem.*, 2011, **83**, 5369–76.

6 H. Li, S. A. Wells, J. E. Jimenez-Roldan, R. A. Römer, Y. Zhao, P. J. Sadler and P. B. O'Connor, *Protein Sci.*, 2012, **21**, 1269–79.

7 C. G. Hartinger, Y. O. Tsybin, J. Fuchser and P. J. Dyson, *Inorg. Chem.*, 2008, **47**, 17–9.

8 M. Scholl, S. Ding, C. W. Lee and R. H. Grubbs, *Org. Lett.*, 1999, **1**, 953–6.

9 M. Black, R. H. B. Mais and P. G. Owston, *Acta Crystallogr. Sect. B Struct. Crystallogr. Cryst. Chem.*, 1969, **25**, 1753–1759.

10 H. J. Cooper, *J. Am. Soc. Mass Spectrom.*, 2005, **16**, 1932–40.

11 D. P. Little, J. P. Speir, M. W. Senko, P. B. O'Connor and F. W. McLafferty, *Anal. Chem.*, 1994, **66**, 2809–15.

12 C. Bleiholder, S. Osburn, T. D. Williams, S. Suhai, M. Van Stipdonk, A. G. Harrison and B. Paizs, *J. Am. Chem. Soc.*, 2008, **130**, 17774–89.

13 A. G. Harrison, A. B. Young, C. Bleiholder, S. Suhai and B. Paizs, *J. Am. Chem. Soc.*, 2006, **128**, 10364–5.

14 E. A. Syrstad and F. Turecek, *J. Am. Soc. Mass Spectrom.*, 2005, **16**, 208–24.
